# Supplementary figures and images for: Characterisation of GLUT4 trafficking in HeLa cells: comparable kinetics and orthologous trafficking mechanisms to 3T3-L1 adipocytes
Source: PeerJ. 2020 Mar 5;8:e8751. doi: 10.7717/peerj.8751 (PMC7060922; doi:10.7717/peerj.8751)

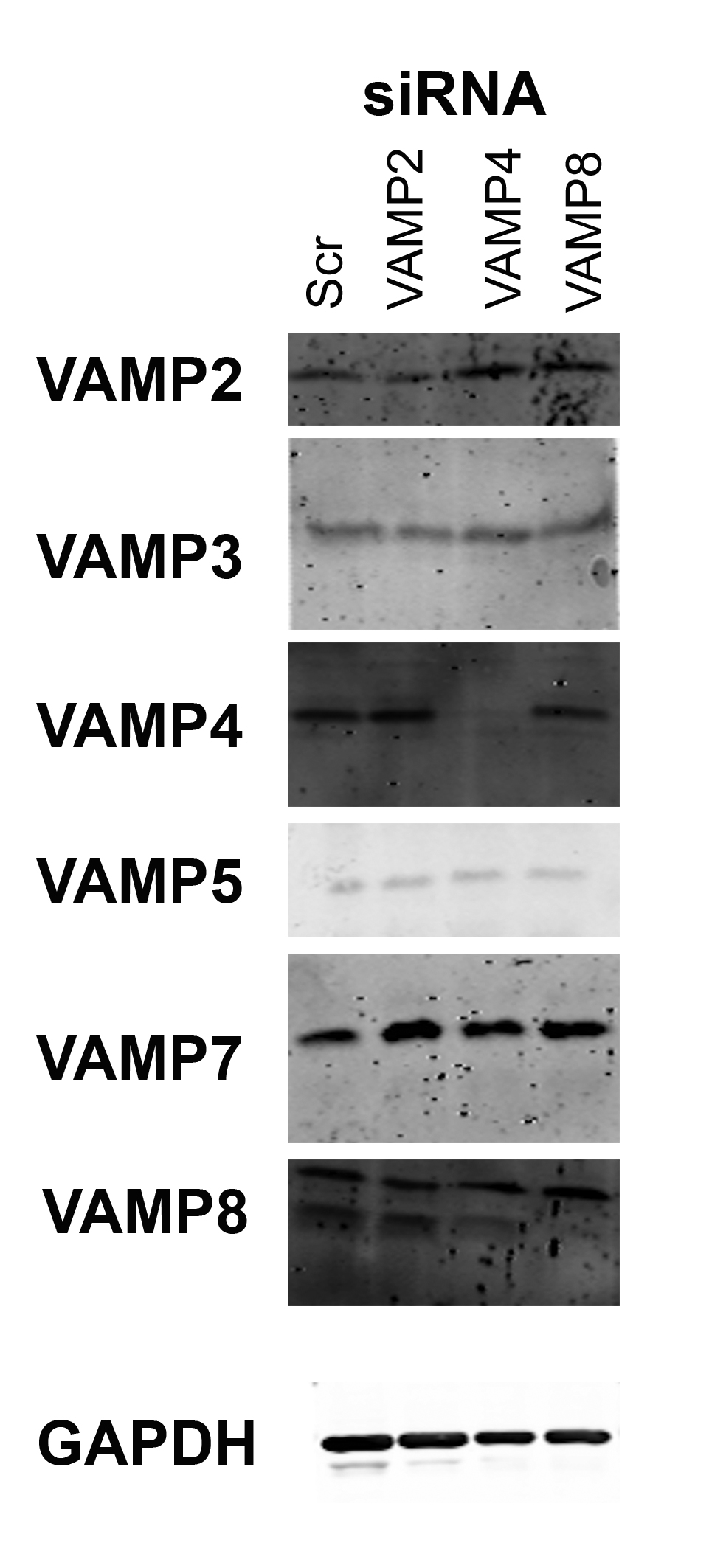

Supplement: Supplemental Information 1 — HA–GLUT4–GFP expressing HeLa cells were transfected with the indicated siRNA SmartPool (SCR = scrambled control) and lysates prepared as described in “Materials and Methods”. Equal amounts of lysate were separate on SDS-PAGE gels and immunoblotted for levels of the proteins indicated on the left of the figure. VAMP2 levels were decreased by 55 + 18% and VAMP4 levels decreased 82 + 9% in n = 4 experiments of this type. [file peerj-08-8751-s001.jpg]

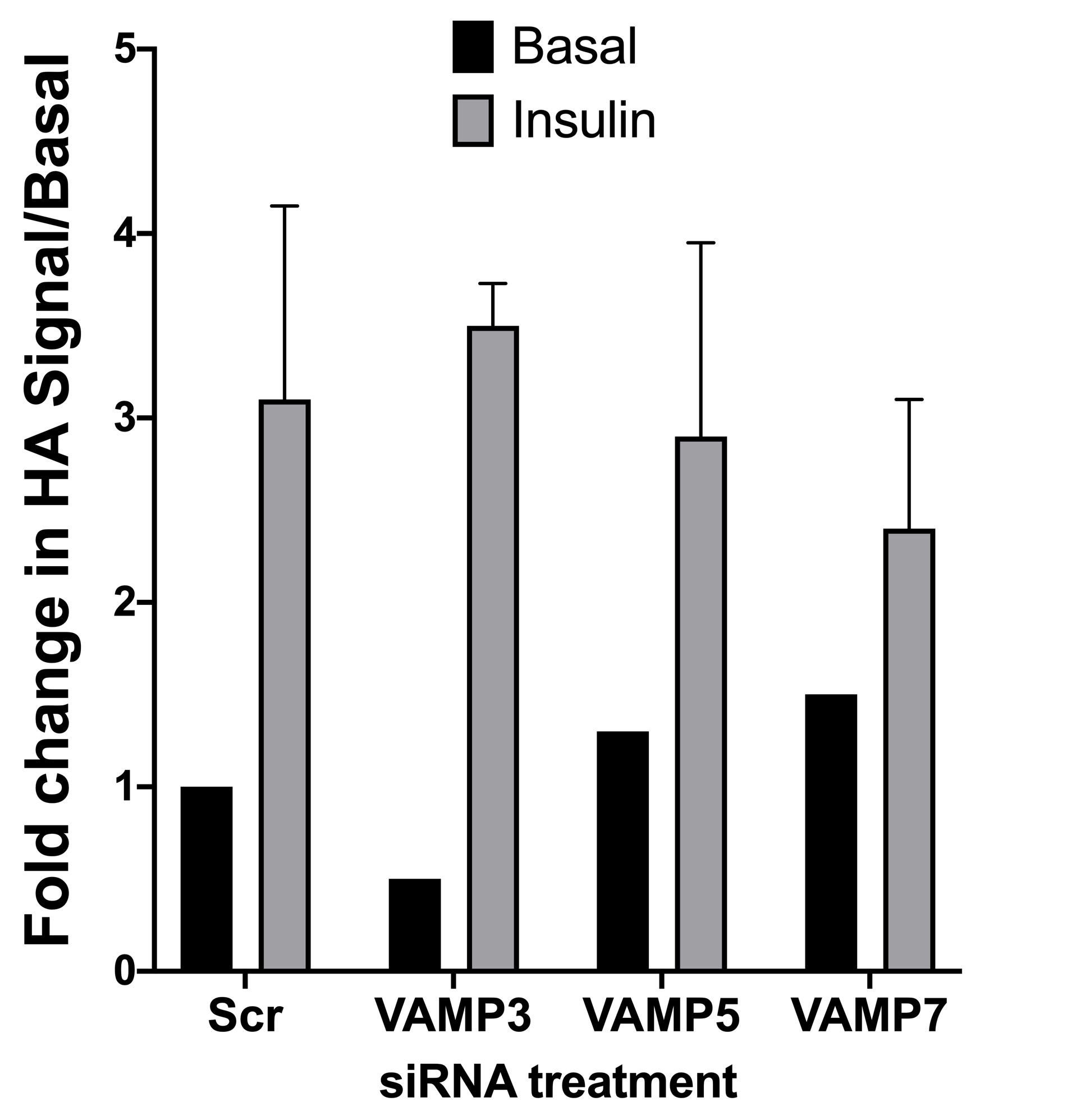

Supplement: Supplemental Information 2 — HeLa cells expressing HA–GLUT4–GFP were grown and transfected with 200 nM scrambled control sequence (Scr), VAMP3, 5 or 7 SMARTpool siRNA as described. Cells were serum-starved (basal) before being treated with or without 1 µM insulin (insulin) for 20 min and cell surface HA staining quantified as outlined in “Materials and Methods”. Values shown are means ± SD of 16 random fields of view, taken from four independent experiments. In each case, insulin induced a statistically significant increase in cell surface GLUT4 staining, but Basal (unstimulated) or insulin-stimulated values did not differ significantly from Scr-treated cells for any of the VAMP knockdowns shown. [file peerj-08-8751-s002.jpg]

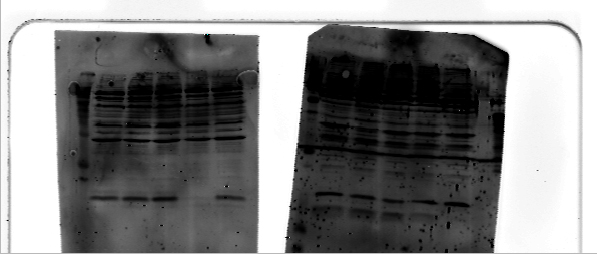

Supplement: Supplemental Information 3 [file peerj-08-8751-s003.zip › Morris et al Data/Data for Supp Fig. 1/VAMP4 and VAMP8.jpg]

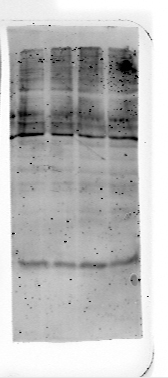

Supplement: Supplemental Information 3 [file peerj-08-8751-s003.zip › Morris et al Data/Data for Supp Fig. 1/VAMP3.jpg]

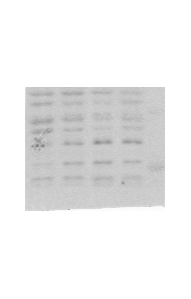

Supplement: Supplemental Information 3 [file peerj-08-8751-s003.zip › Morris et al Data/Data for Supp Fig. 1/VAMP5.jpg]

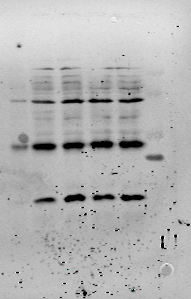

Supplement: Supplemental Information 3 [file peerj-08-8751-s003.zip › Morris et al Data/Data for Supp Fig. 1/VAMP7.jpg]

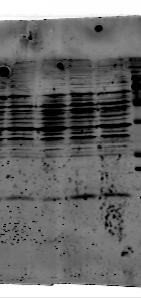

Supplement: Supplemental Information 3 [file peerj-08-8751-s003.zip › Morris et al Data/Data for Supp Fig. 1/VAMP2 blot.jpg]

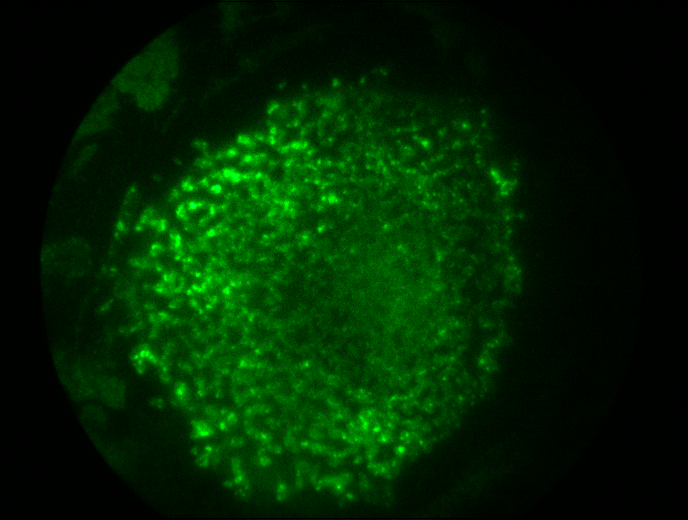

Supplement: Supplemental Information 3 [file peerj-08-8751-s003.zip › Morris et al Data/images of Figure 2/t9 (60).bmp]

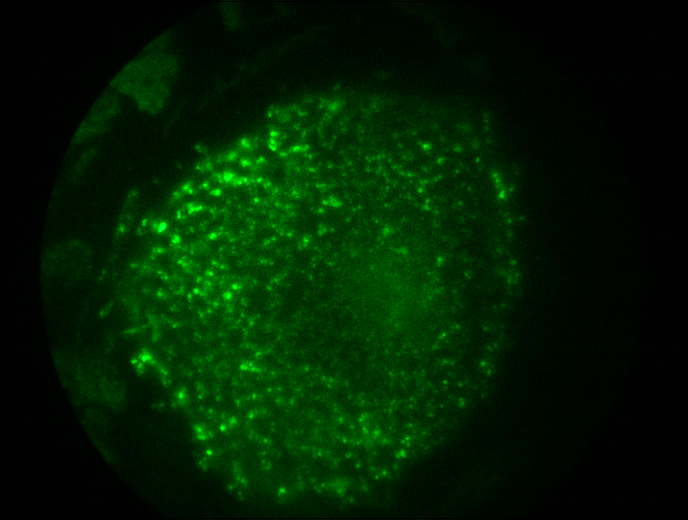

Supplement: Supplemental Information 3 [file peerj-08-8751-s003.zip › Morris et al Data/images of Figure 2/t3 (60).bmp]

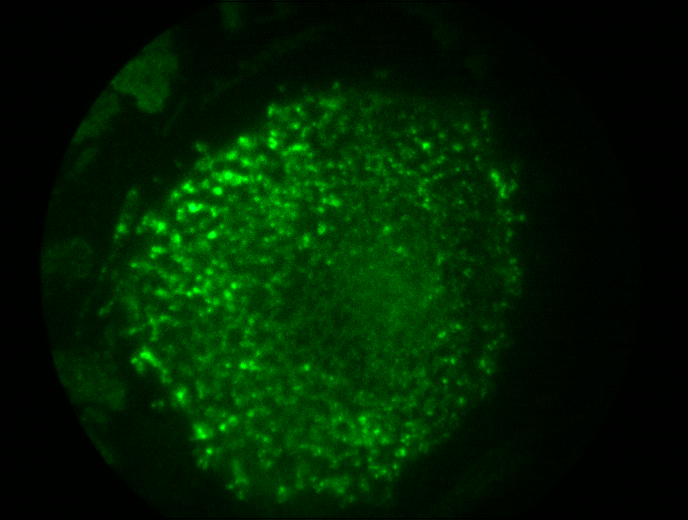

Supplement: Supplemental Information 3 [file peerj-08-8751-s003.zip › Morris et al Data/images of Figure 2/t5 (60).bmp]

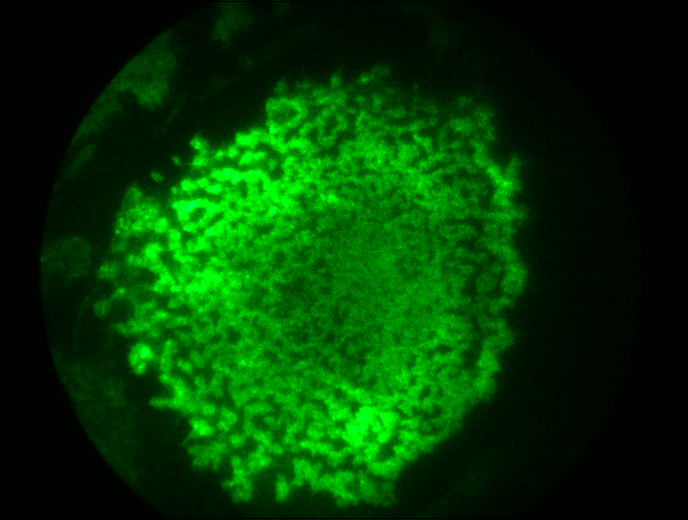

Supplement: Supplemental Information 3 [file peerj-08-8751-s003.zip › Morris et al Data/images of Figure 2/t25 (60).bmp]

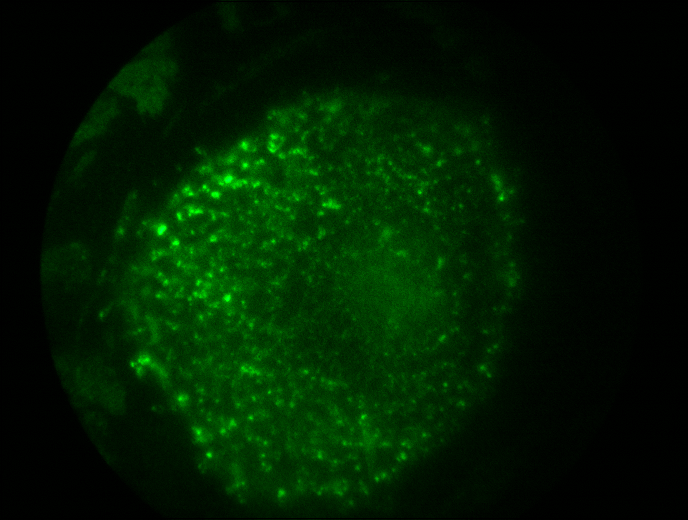

Supplement: Supplemental Information 3 [file peerj-08-8751-s003.zip › Morris et al Data/images of Figure 2/t1 (60).bmp]

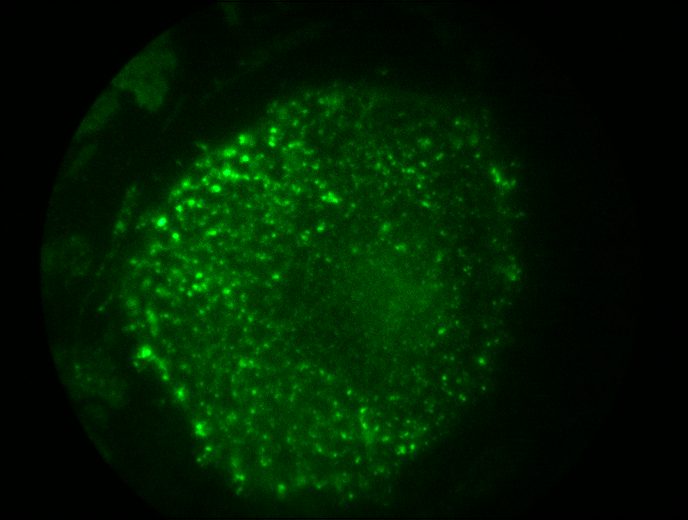

Supplement: Supplemental Information 3 [file peerj-08-8751-s003.zip › Morris et al Data/images of Figure 2/t0 (60).bmp]

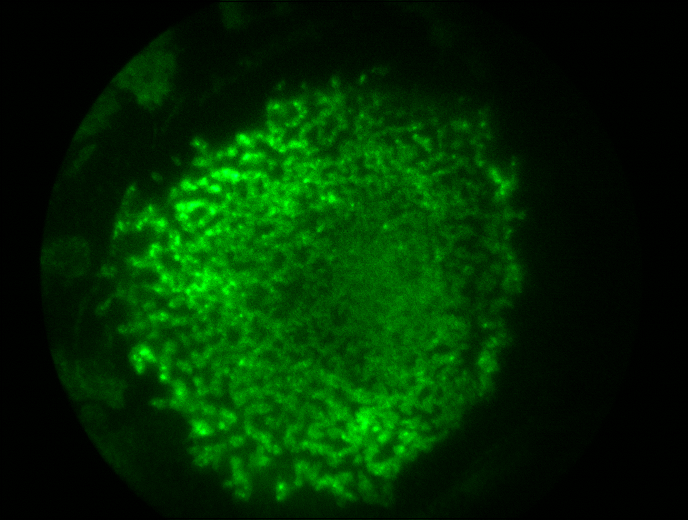

Supplement: Supplemental Information 3 [file peerj-08-8751-s003.zip › Morris et al Data/images of Figure 2/t15 (60).bmp]

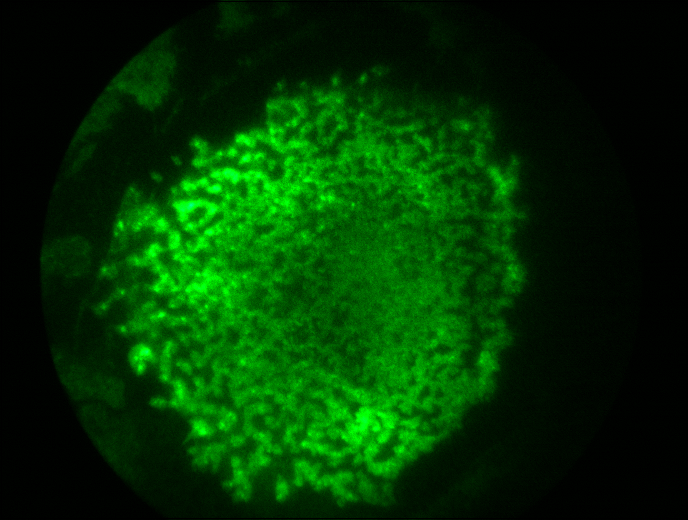

Supplement: Supplemental Information 3 [file peerj-08-8751-s003.zip › Morris et al Data/images of Figure 2/t20 (60).bmp]

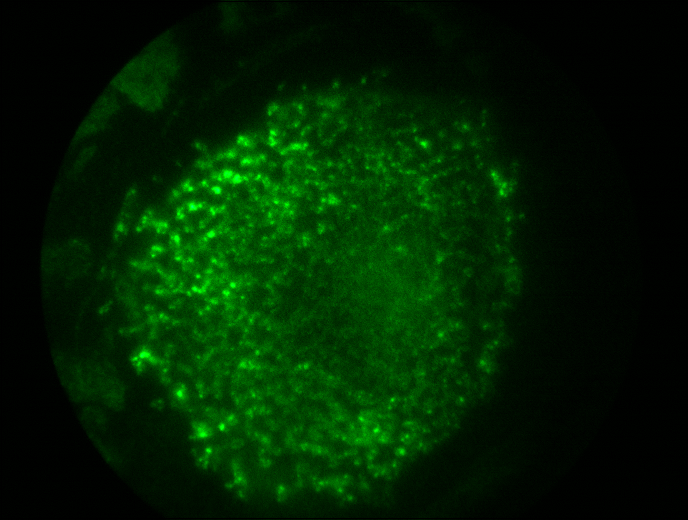

Supplement: Supplemental Information 3 [file peerj-08-8751-s003.zip › Morris et al Data/images of Figure 2/t7 (60).bmp]

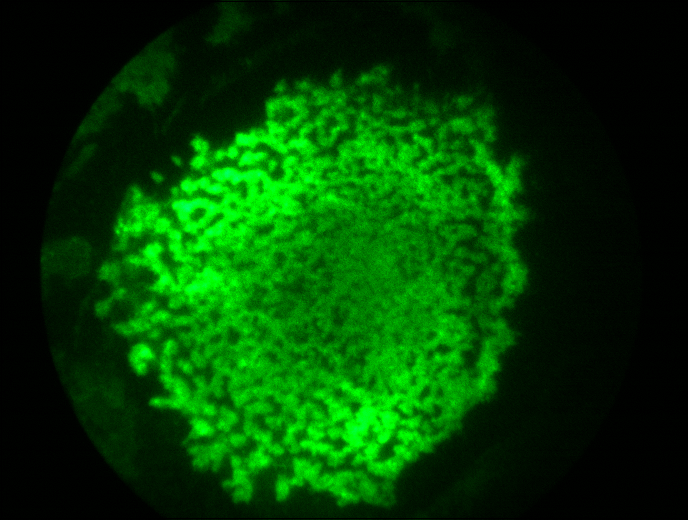

Supplement: Supplemental Information 3 [file peerj-08-8751-s003.zip › Morris et al Data/images of Figure 2/t30 (60).bmp]

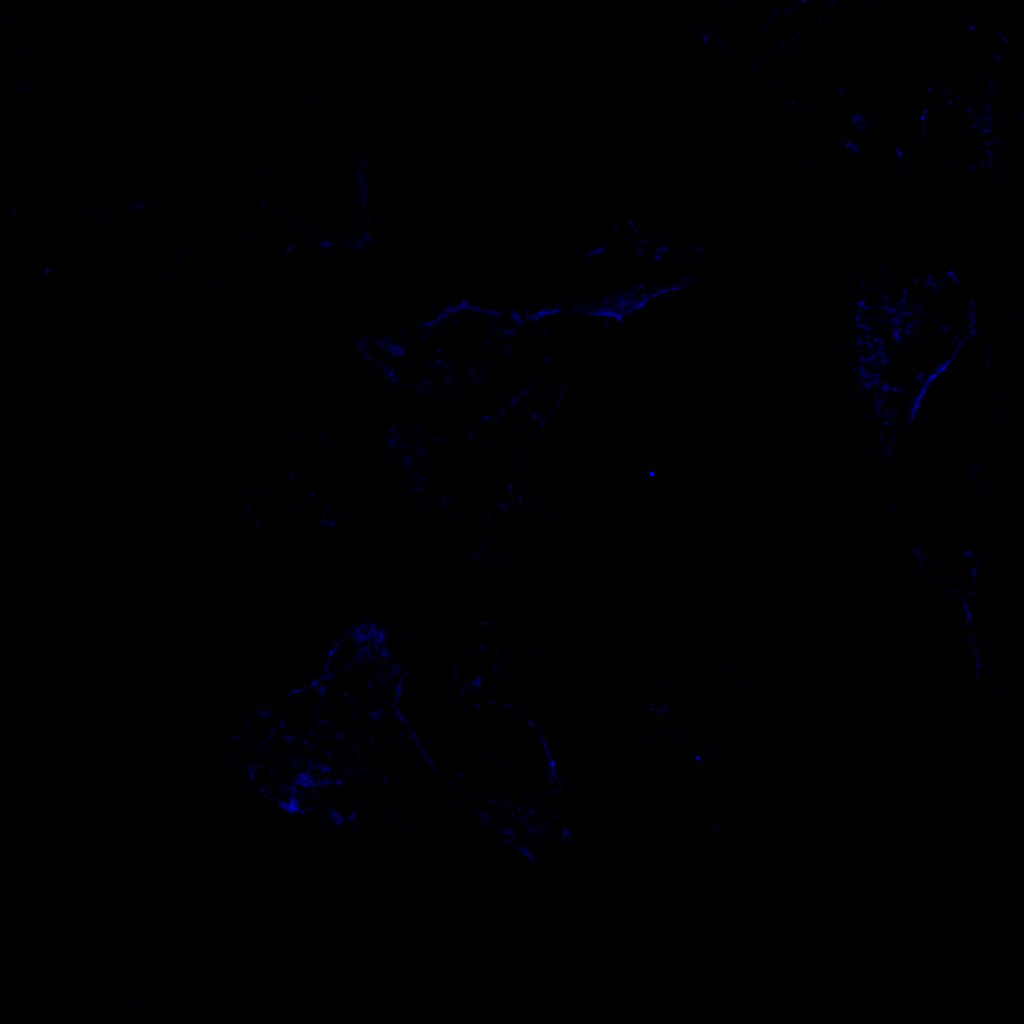

Supplement: Supplemental Information 3 [file peerj-08-8751-s003.zip › Morris et al Data/images for Figure 1/basal1c blue.jpg]

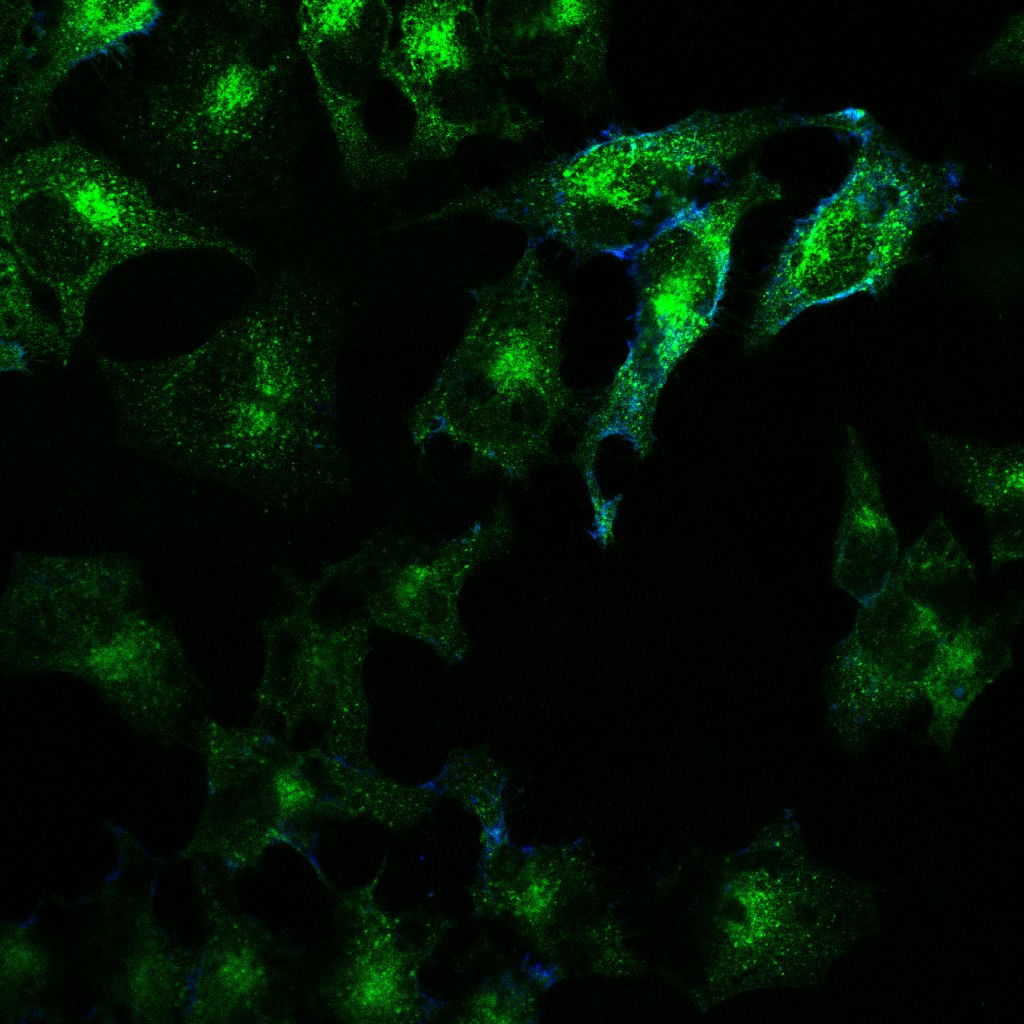

Supplement: Supplemental Information 3 [file peerj-08-8751-s003.zip › Morris et al Data/images for Figure 1/basal1e overlay.jpg]

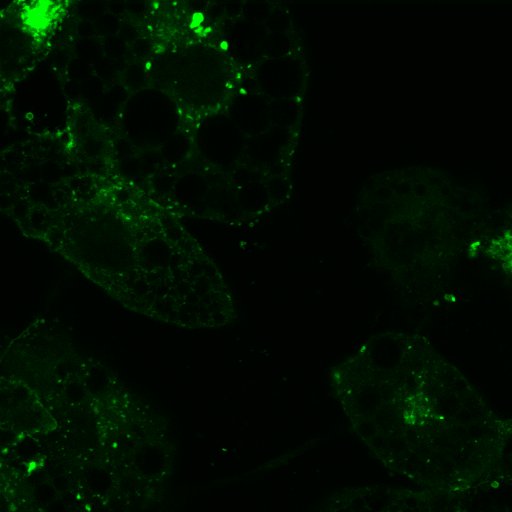

Supplement: Supplemental Information 3 [file peerj-08-8751-s003.zip › Morris et al Data/images for Figure 1/Ha-G4-GFPBasal4 green.jpg]

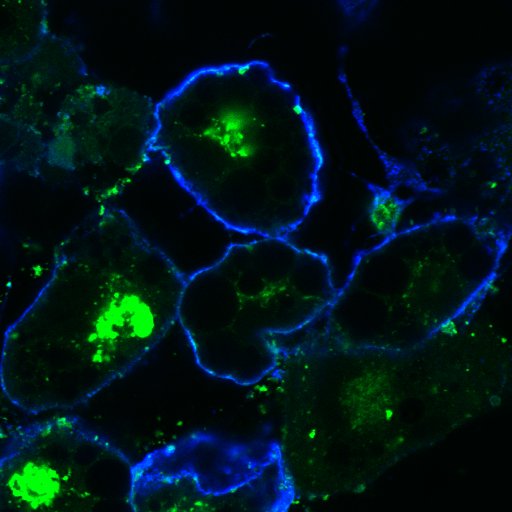

Supplement: Supplemental Information 3 [file peerj-08-8751-s003.zip › Morris et al Data/images for Figure 1/Ha-G4-GFPInsulin4 comp.jpg]

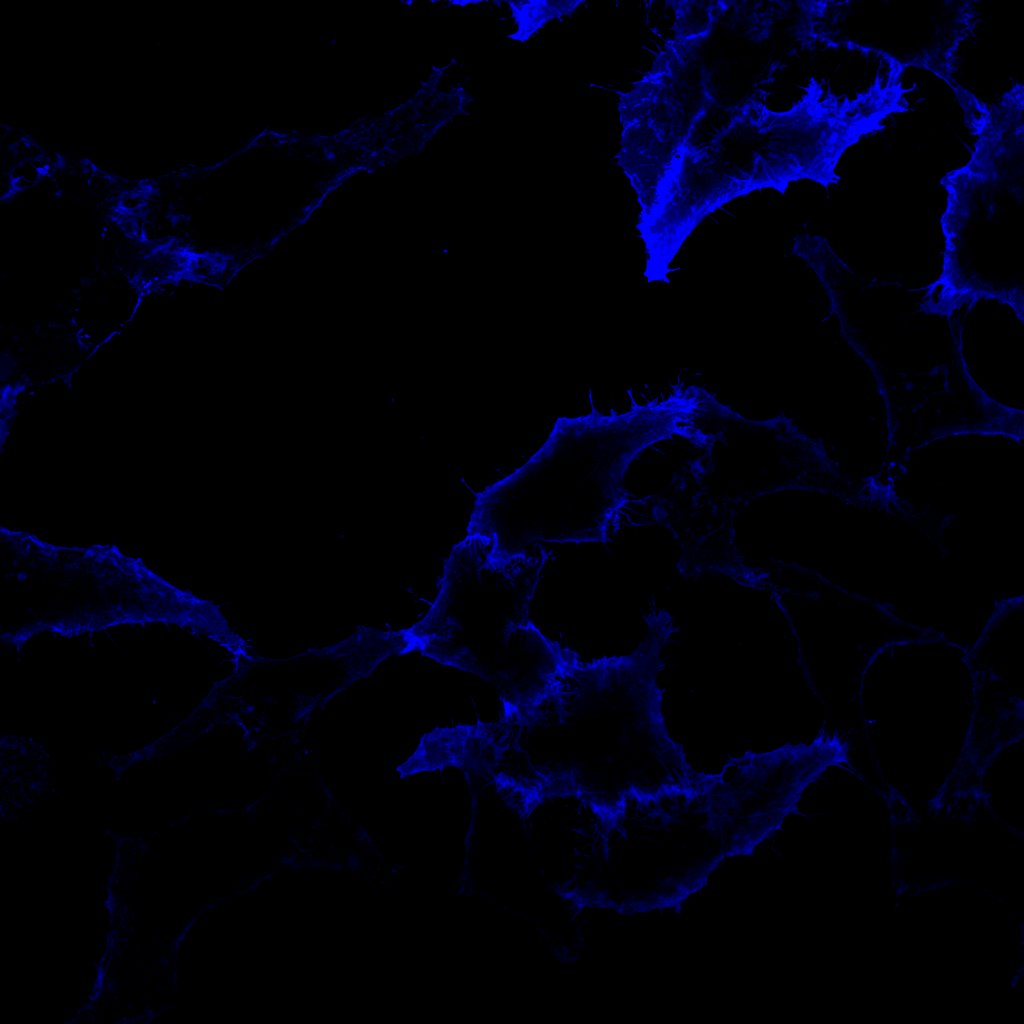

Supplement: Supplemental Information 3 [file peerj-08-8751-s003.zip › Morris et al Data/images for Figure 1/Ins1b blue.jpg]

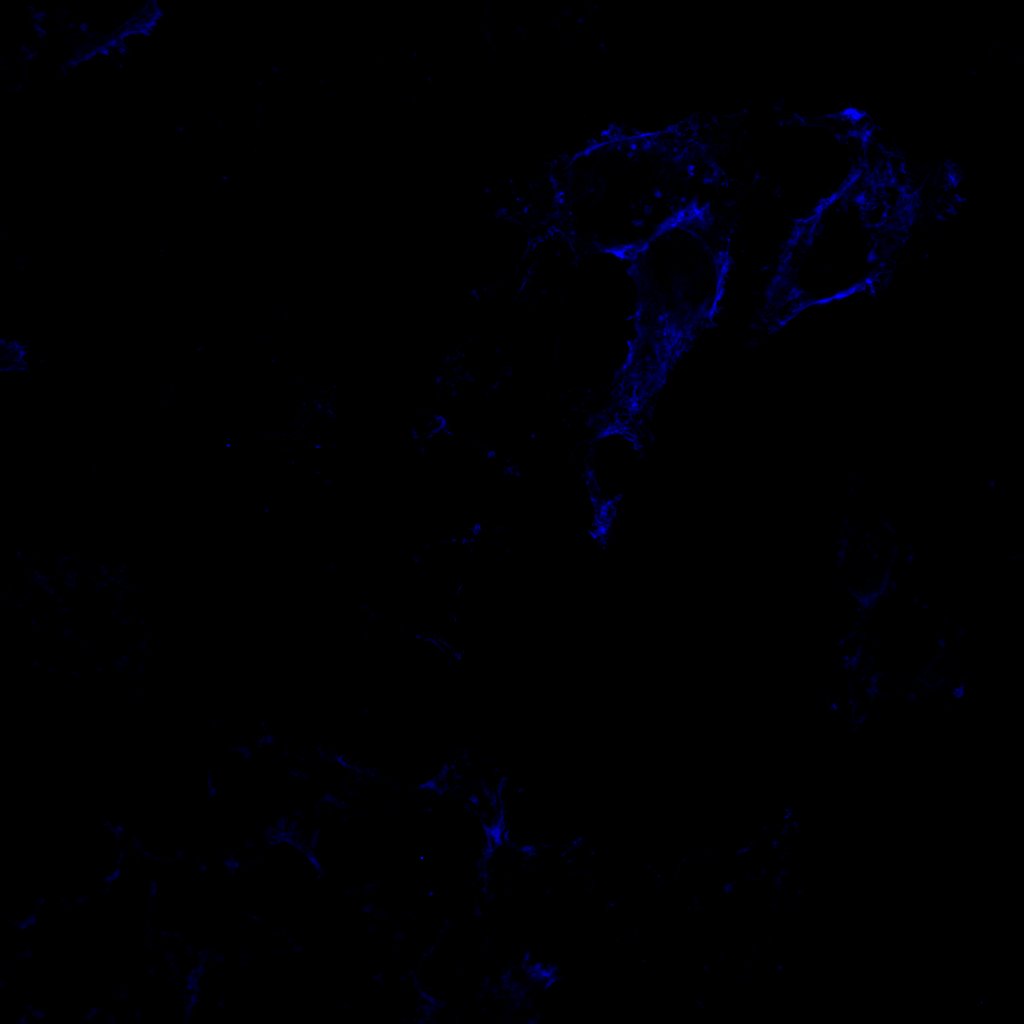

Supplement: Supplemental Information 3 [file peerj-08-8751-s003.zip › Morris et al Data/images for Figure 1/basal1e blue.jpg]

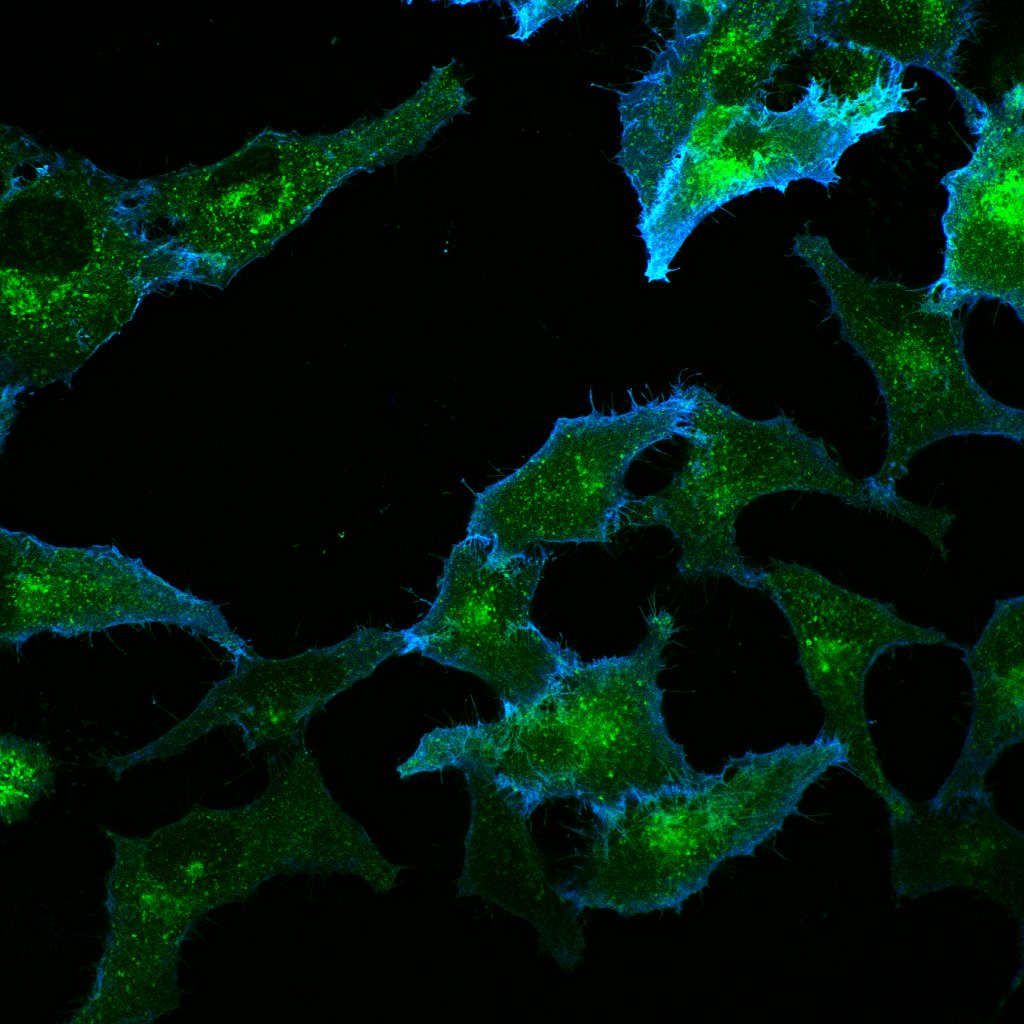

Supplement: Supplemental Information 3 [file peerj-08-8751-s003.zip › Morris et al Data/images for Figure 1/Composite ins.jpg]

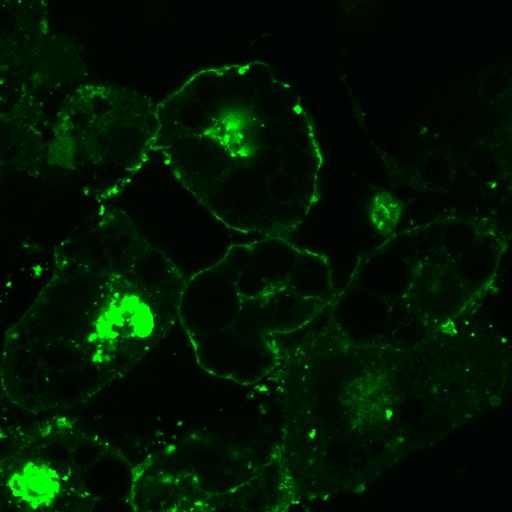

Supplement: Supplemental Information 3 [file peerj-08-8751-s003.zip › Morris et al Data/images for Figure 1/Ha-G4-GFPInsulin4 green.jpg]

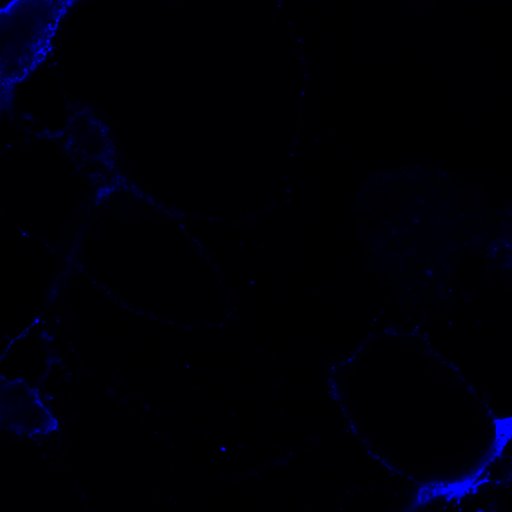

Supplement: Supplemental Information 3 [file peerj-08-8751-s003.zip › Morris et al Data/images for Figure 1/Ha-G4-GFPBasal4 blue.jpg]

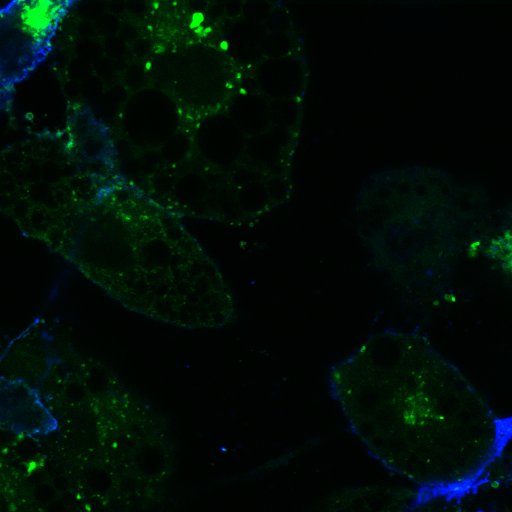

Supplement: Supplemental Information 3 [file peerj-08-8751-s003.zip › Morris et al Data/images for Figure 1/Ha-G4-GFPBasal4 composite.jpg]

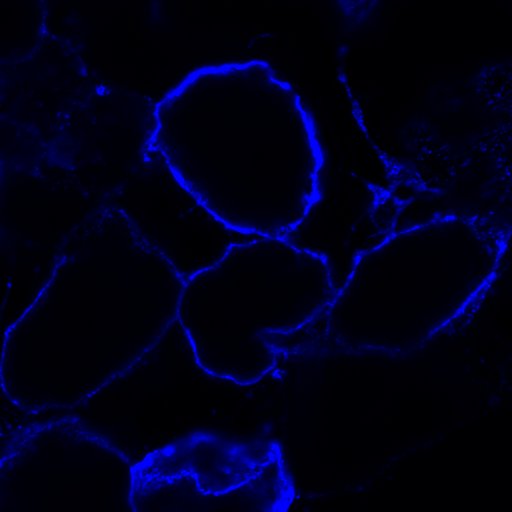

Supplement: Supplemental Information 3 [file peerj-08-8751-s003.zip › Morris et al Data/images for Figure 1/Ha-G4-GFPInsulin4 blue.jpg]

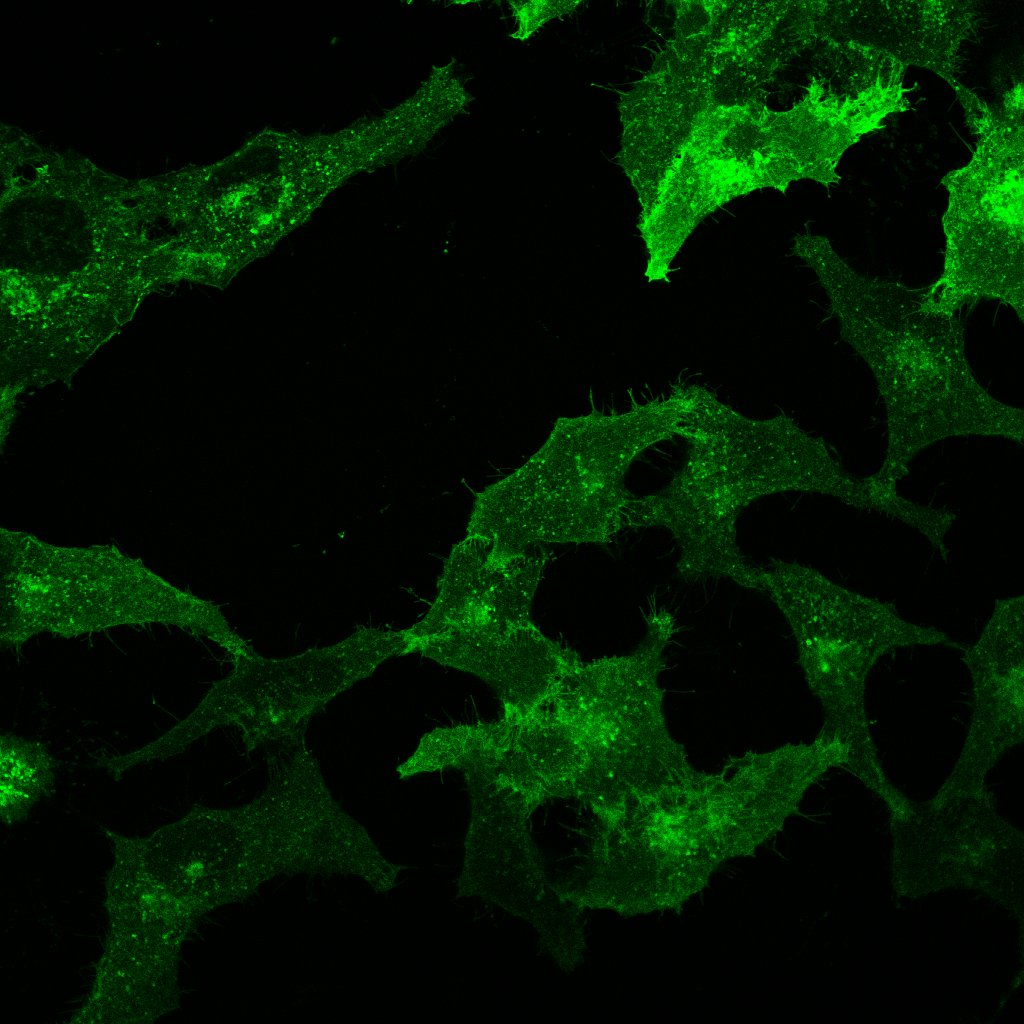

Supplement: Supplemental Information 3 [file peerj-08-8751-s003.zip › Morris et al Data/images for Figure 1/Ins1b green.jpg]

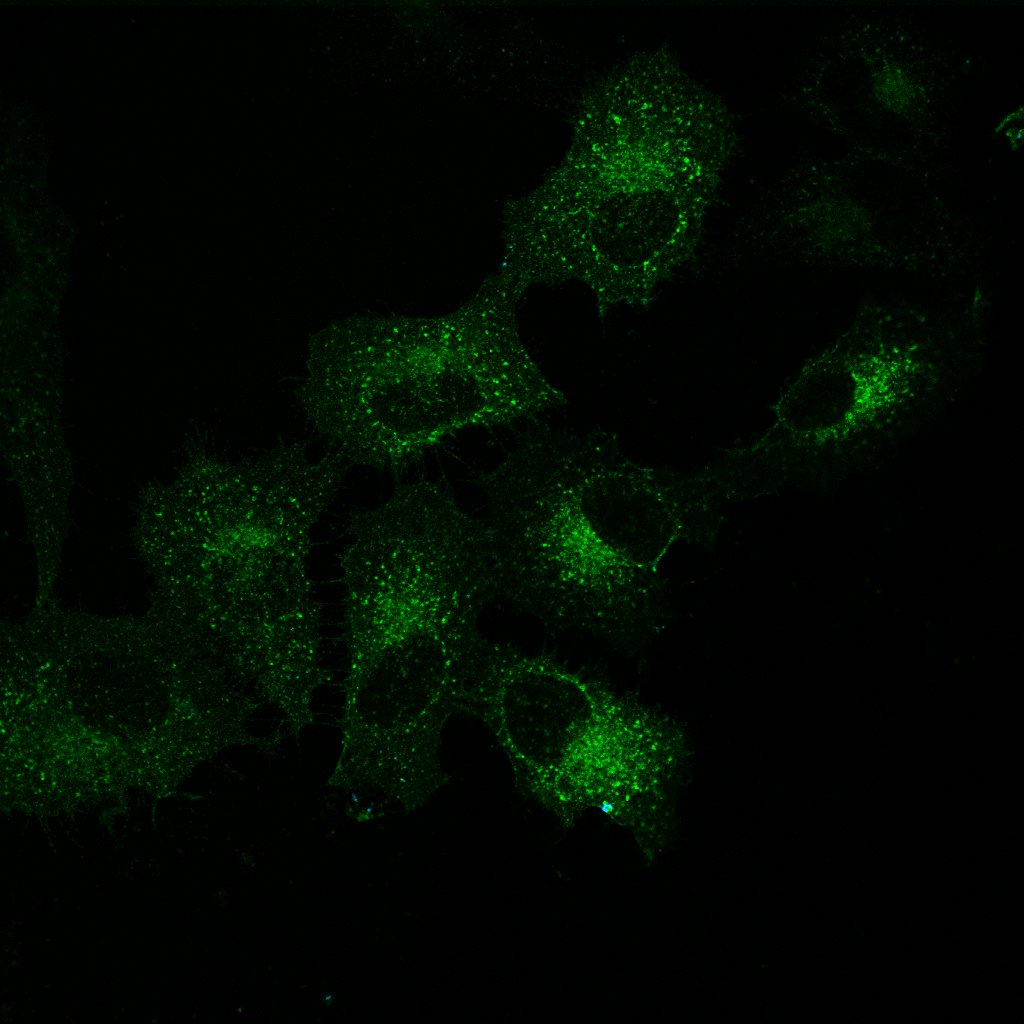

Supplement: Supplemental Information 3 [file peerj-08-8751-s003.zip › Morris et al Data/images for Figure 4/SCRbasal1b0 comp.jpg]

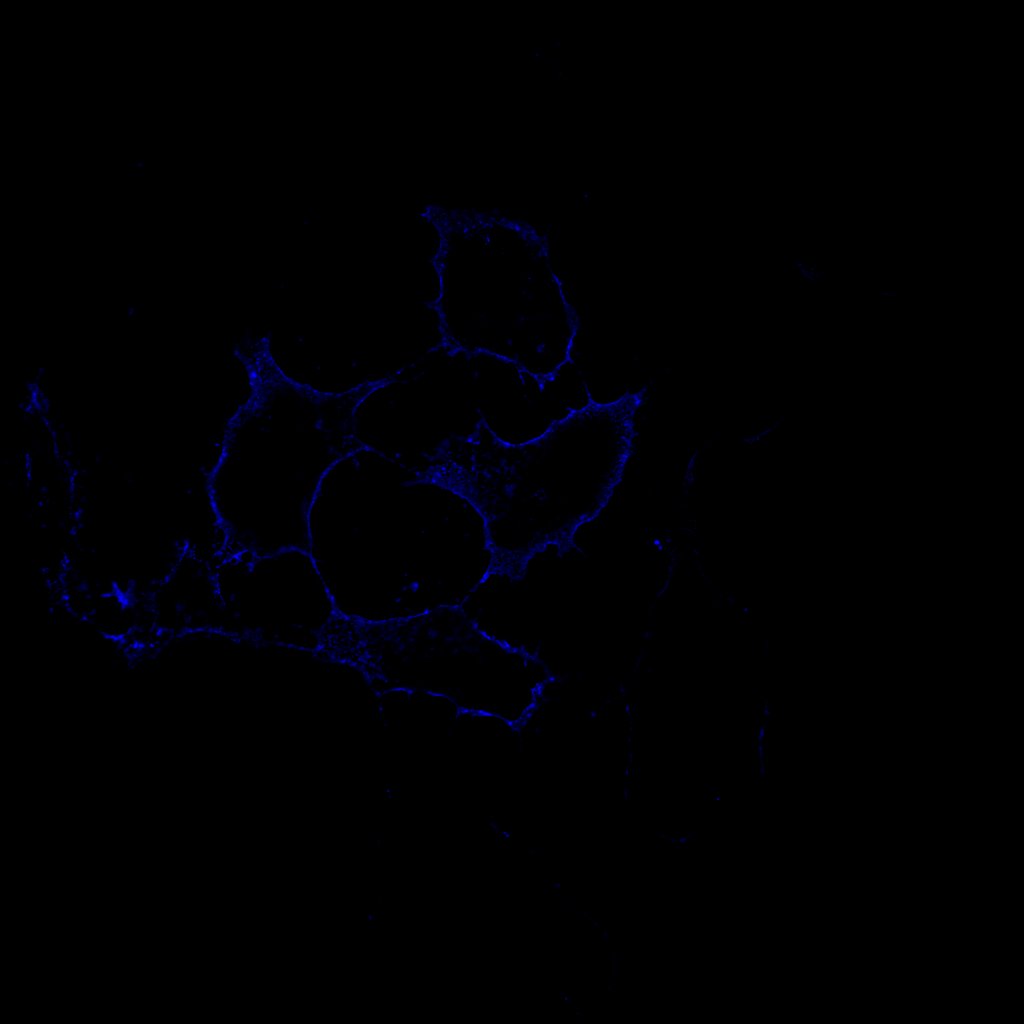

Supplement: Supplemental Information 3 [file peerj-08-8751-s003.zip › Morris et al Data/images for Figure 4/V8Ins1c blue.jpg]

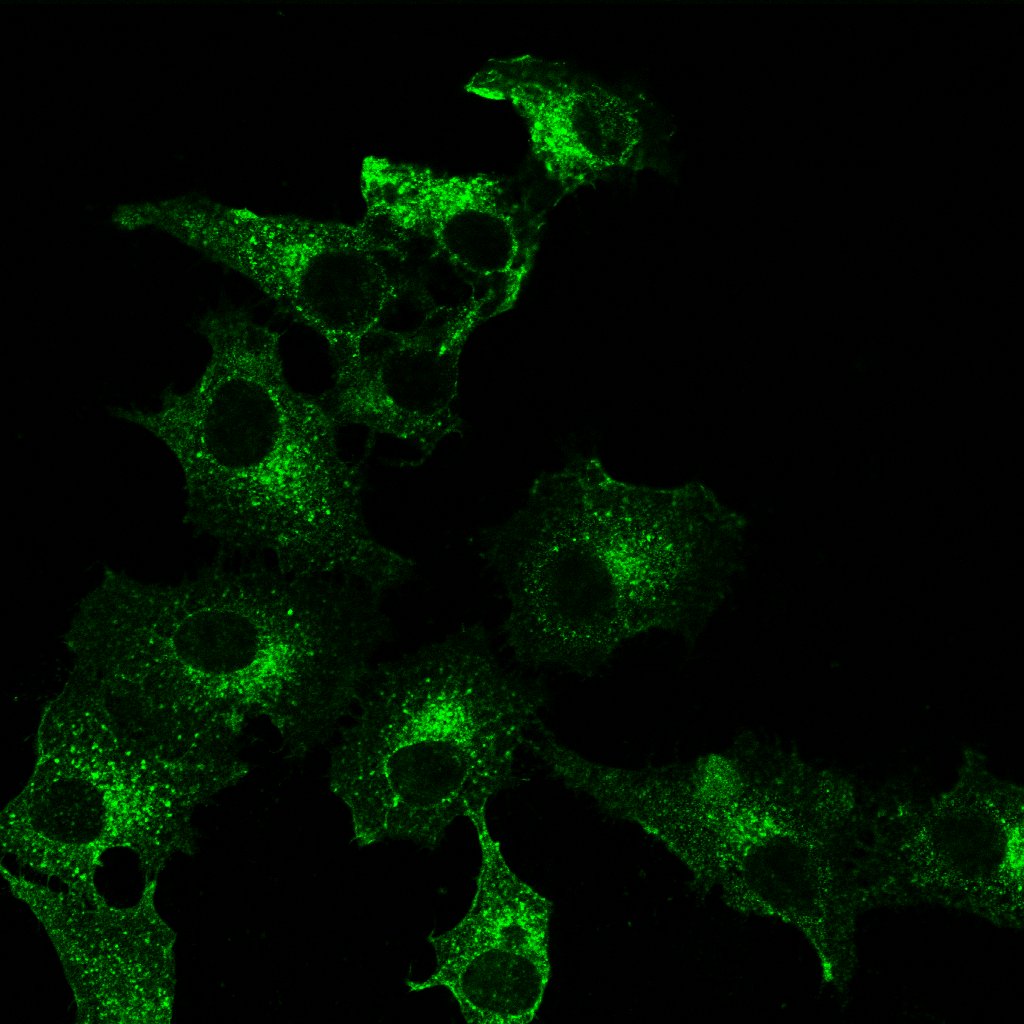

Supplement: Supplemental Information 3 [file peerj-08-8751-s003.zip › Morris et al Data/images for Figure 4/V4basal1c green.jpg]

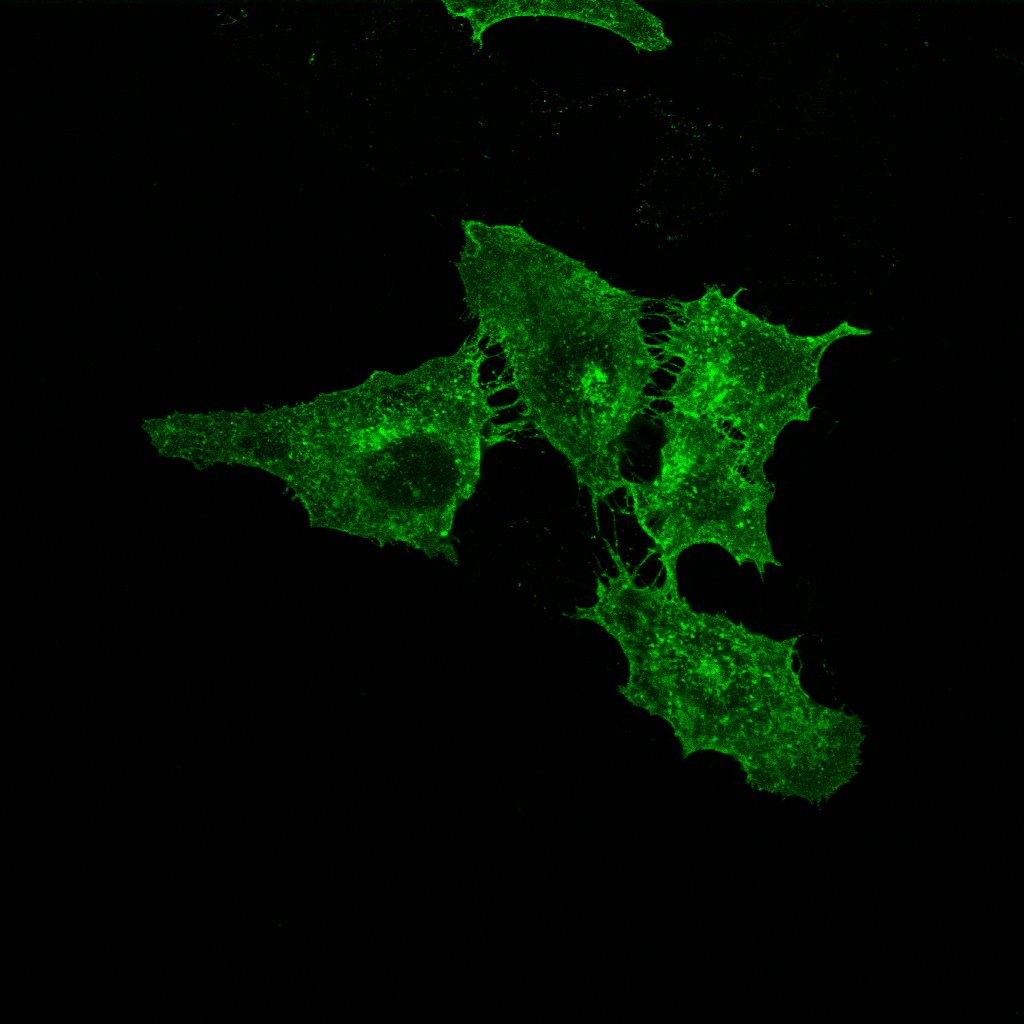

Supplement: Supplemental Information 3 [file peerj-08-8751-s003.zip › Morris et al Data/images for Figure 4/V2basal1a green.jpg]

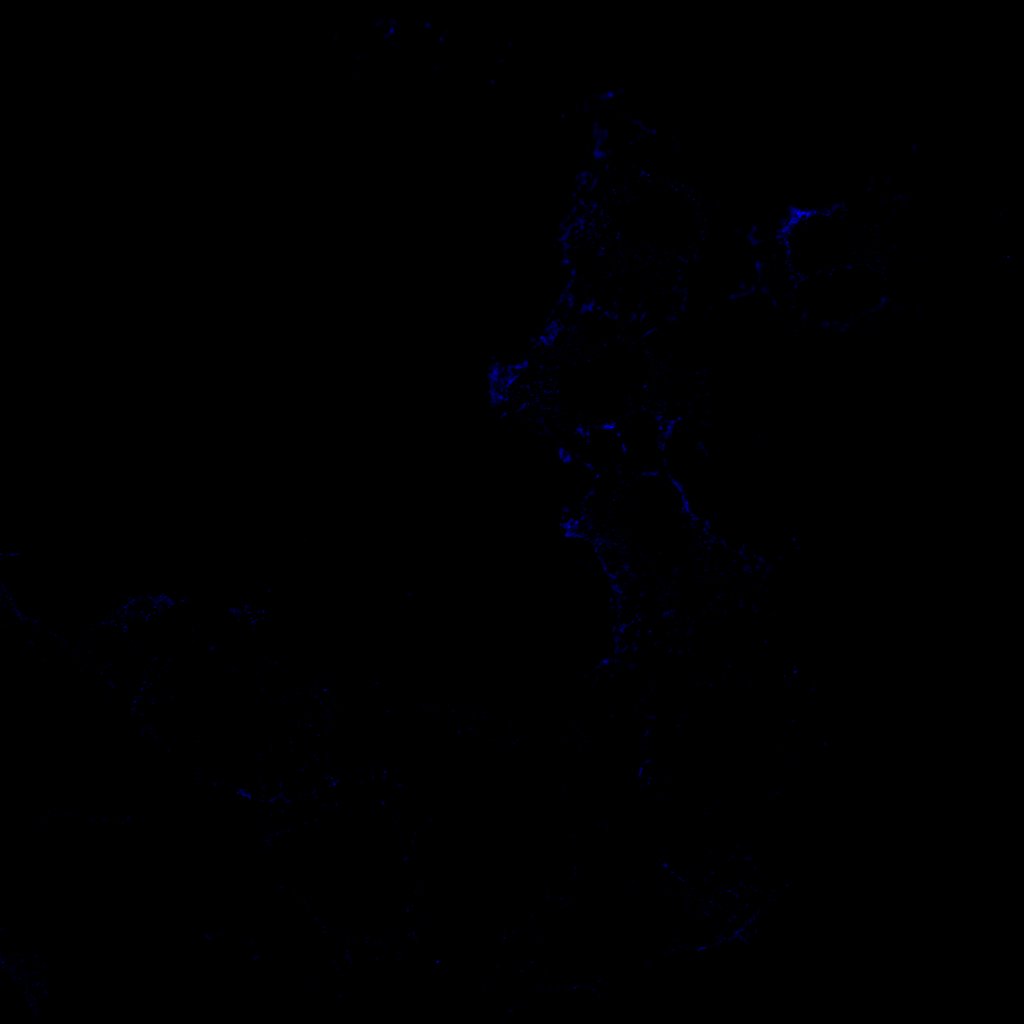

Supplement: Supplemental Information 3 [file peerj-08-8751-s003.zip › Morris et al Data/images for Figure 4/v8basal2b blue.jpg]

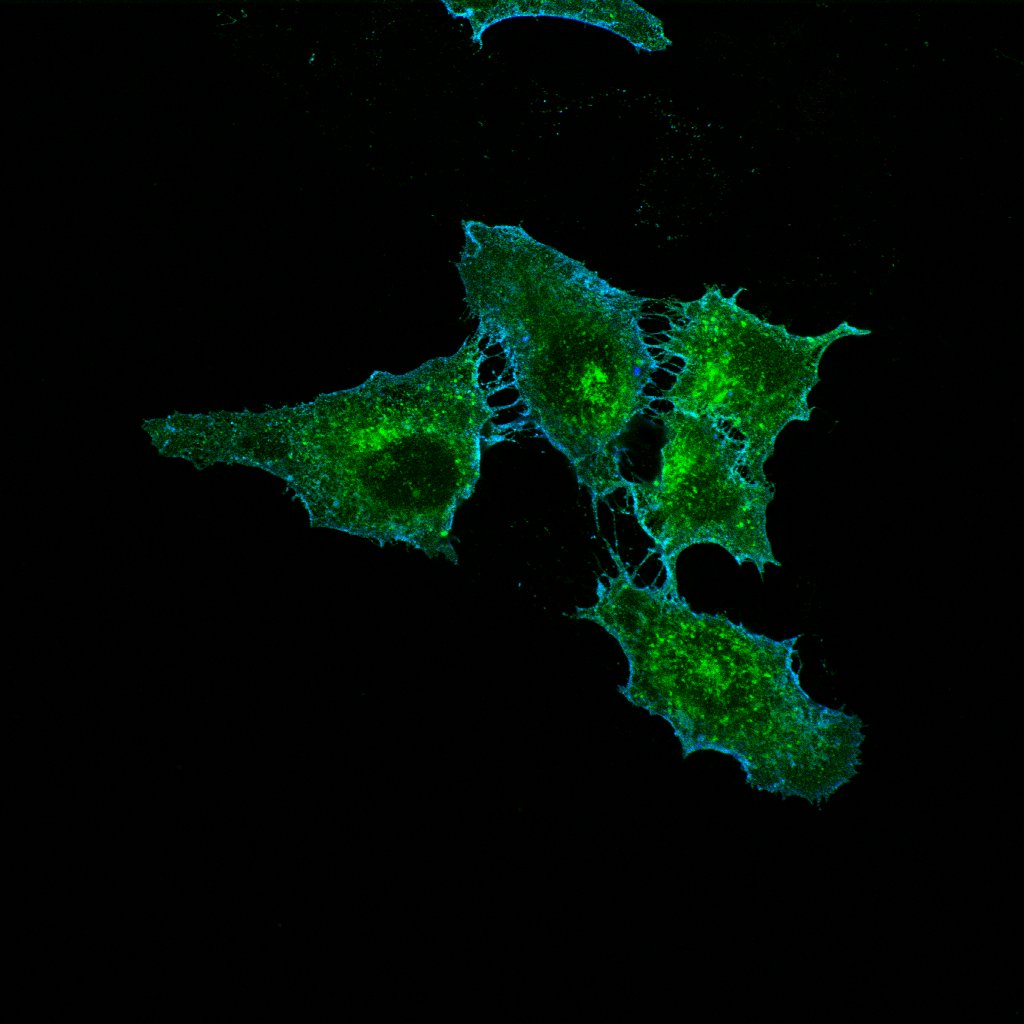

Supplement: Supplemental Information 3 [file peerj-08-8751-s003.zip › Morris et al Data/images for Figure 4/V2basal1a comp.jpg]

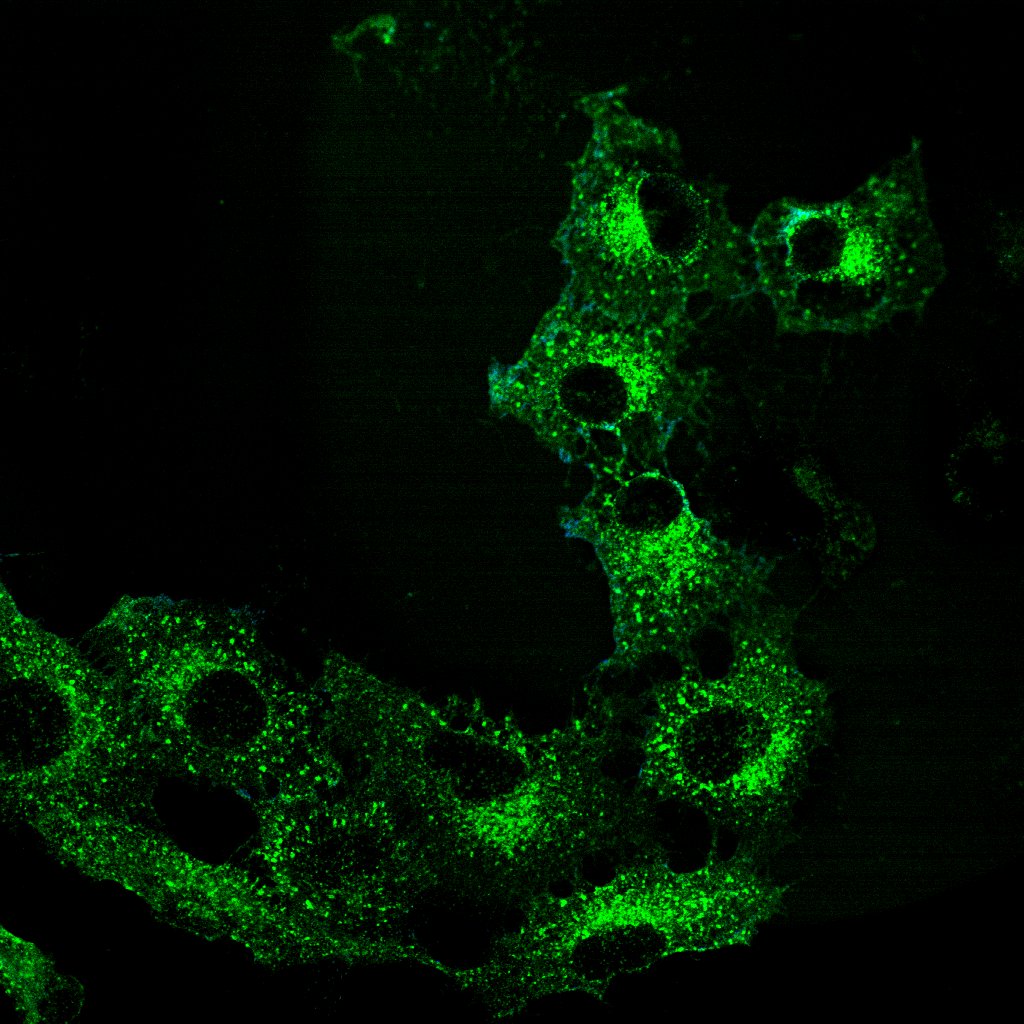

Supplement: Supplemental Information 3 [file peerj-08-8751-s003.zip › Morris et al Data/images for Figure 4/v8basal2b merge.jpg]

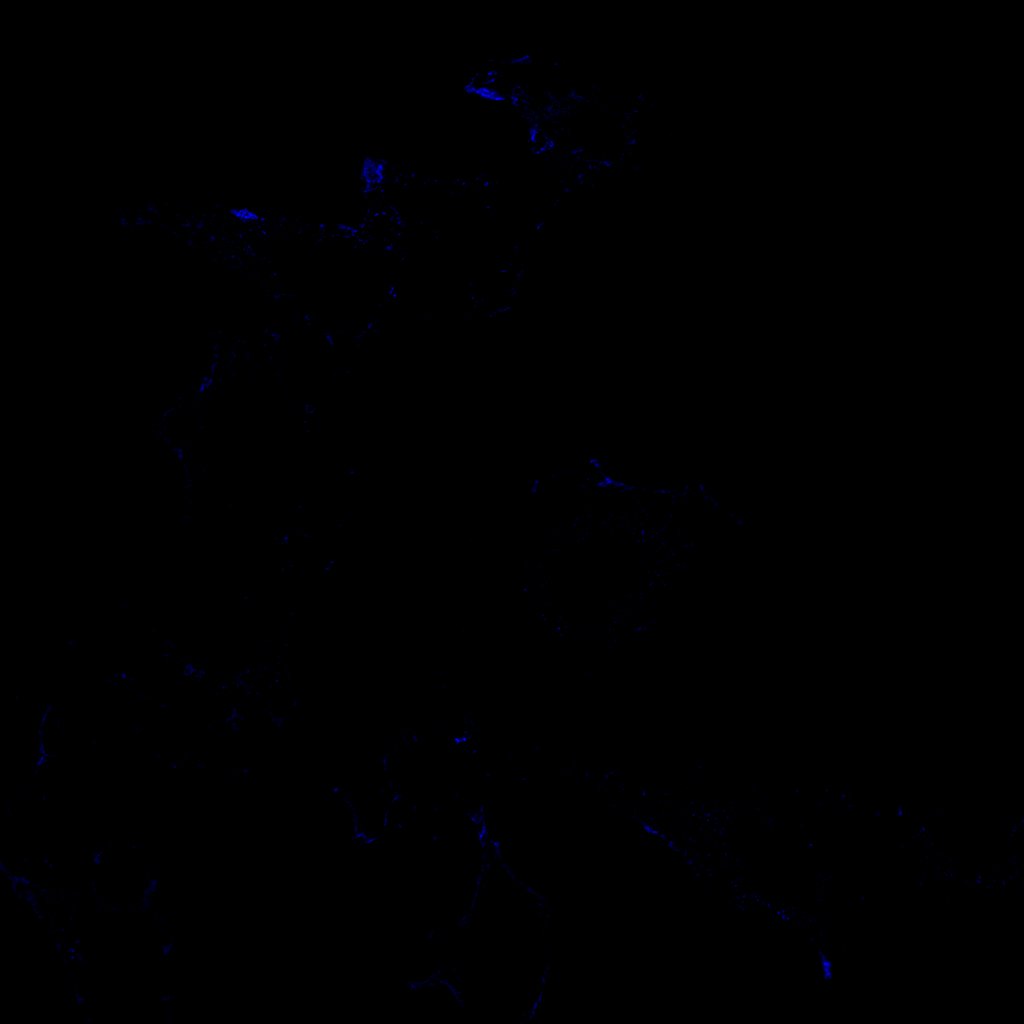

Supplement: Supplemental Information 3 [file peerj-08-8751-s003.zip › Morris et al Data/images for Figure 4/V4basal1c blue.jpg]

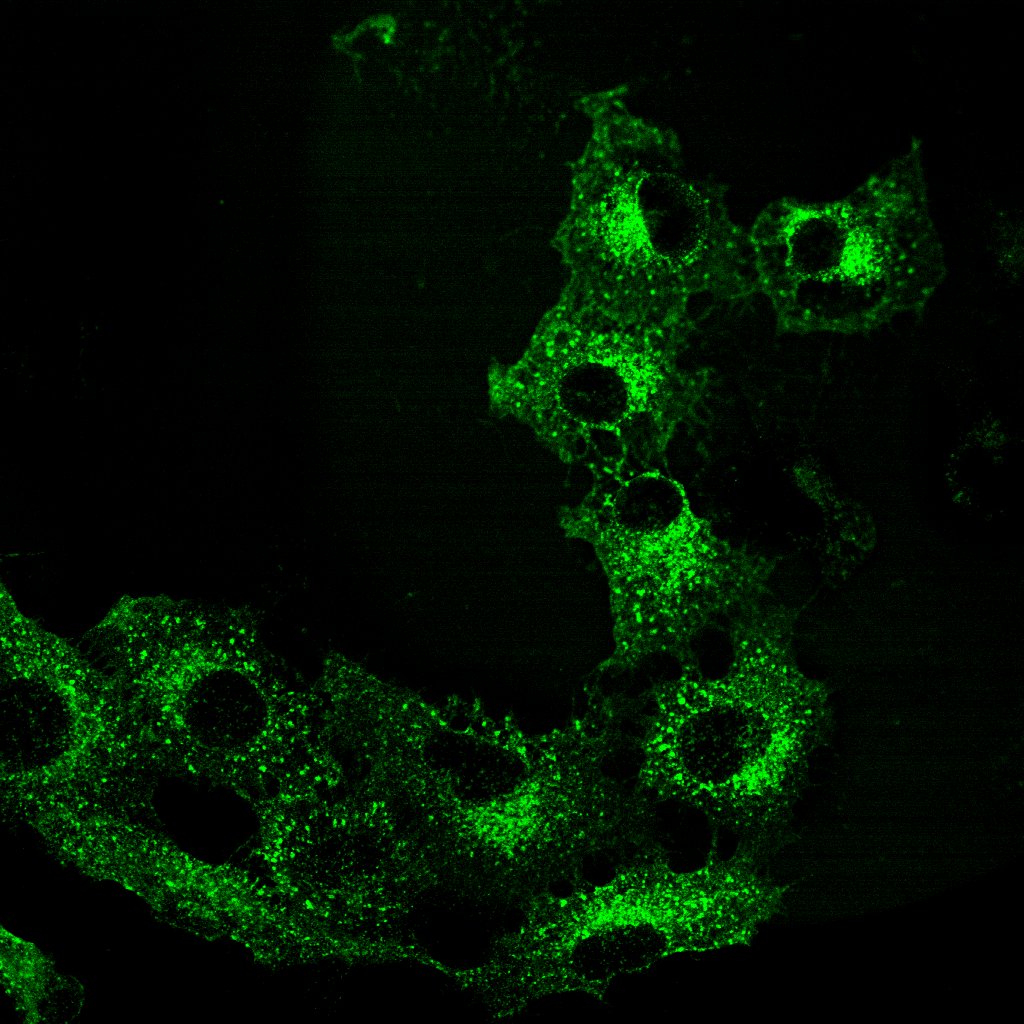

Supplement: Supplemental Information 3 [file peerj-08-8751-s003.zip › Morris et al Data/images for Figure 4/v8basal2b green.jpg]

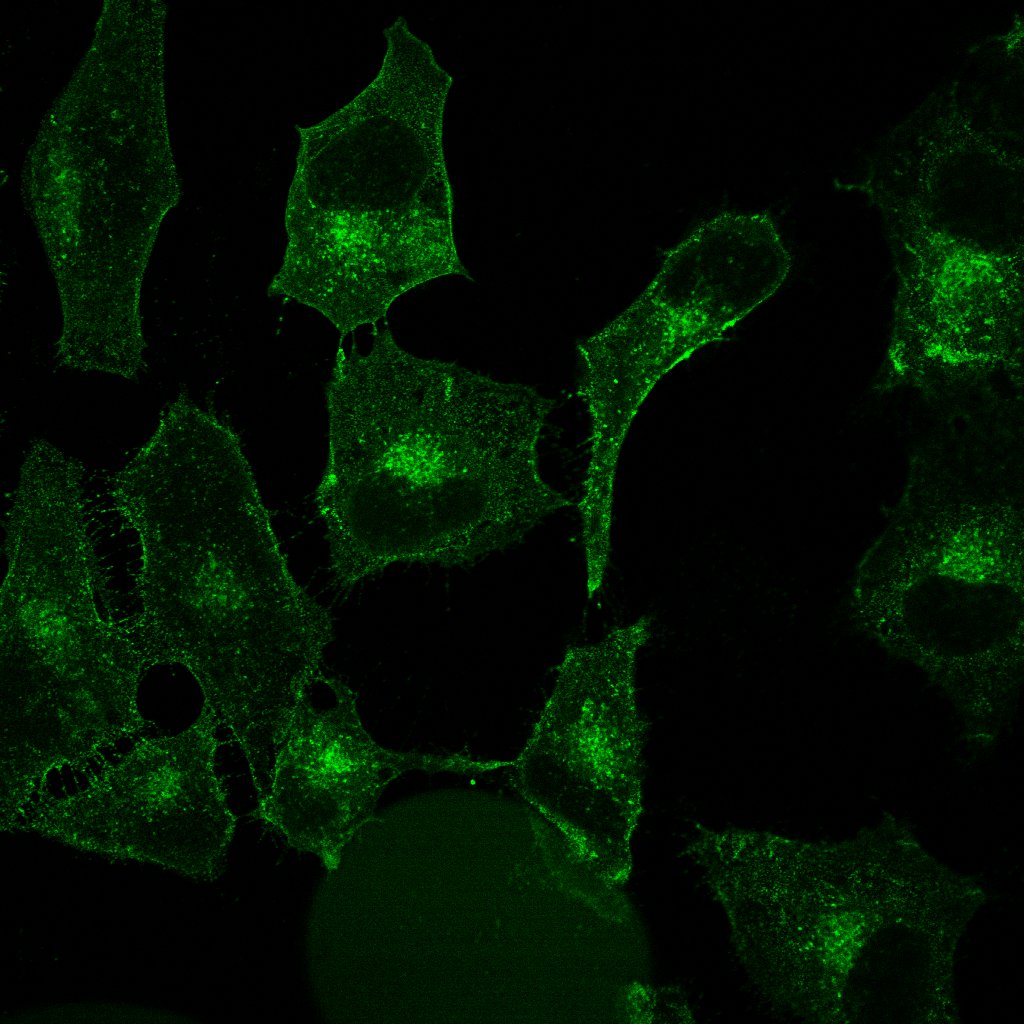

Supplement: Supplemental Information 3 [file peerj-08-8751-s003.zip › Morris et al Data/images for Figure 4/SCRIns1a green.jpg]

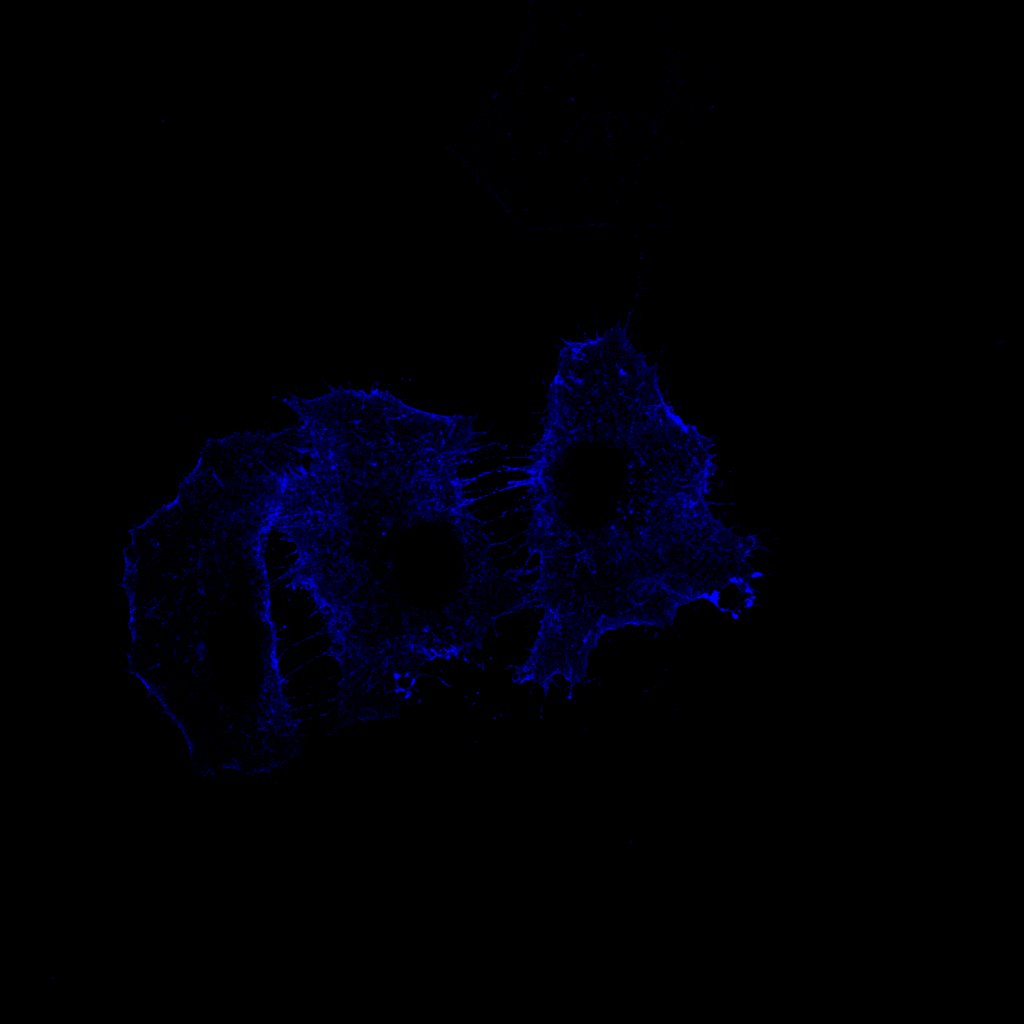

Supplement: Supplemental Information 3 [file peerj-08-8751-s003.zip › Morris et al Data/images for Figure 4/V2Ins1b blue.jpg]

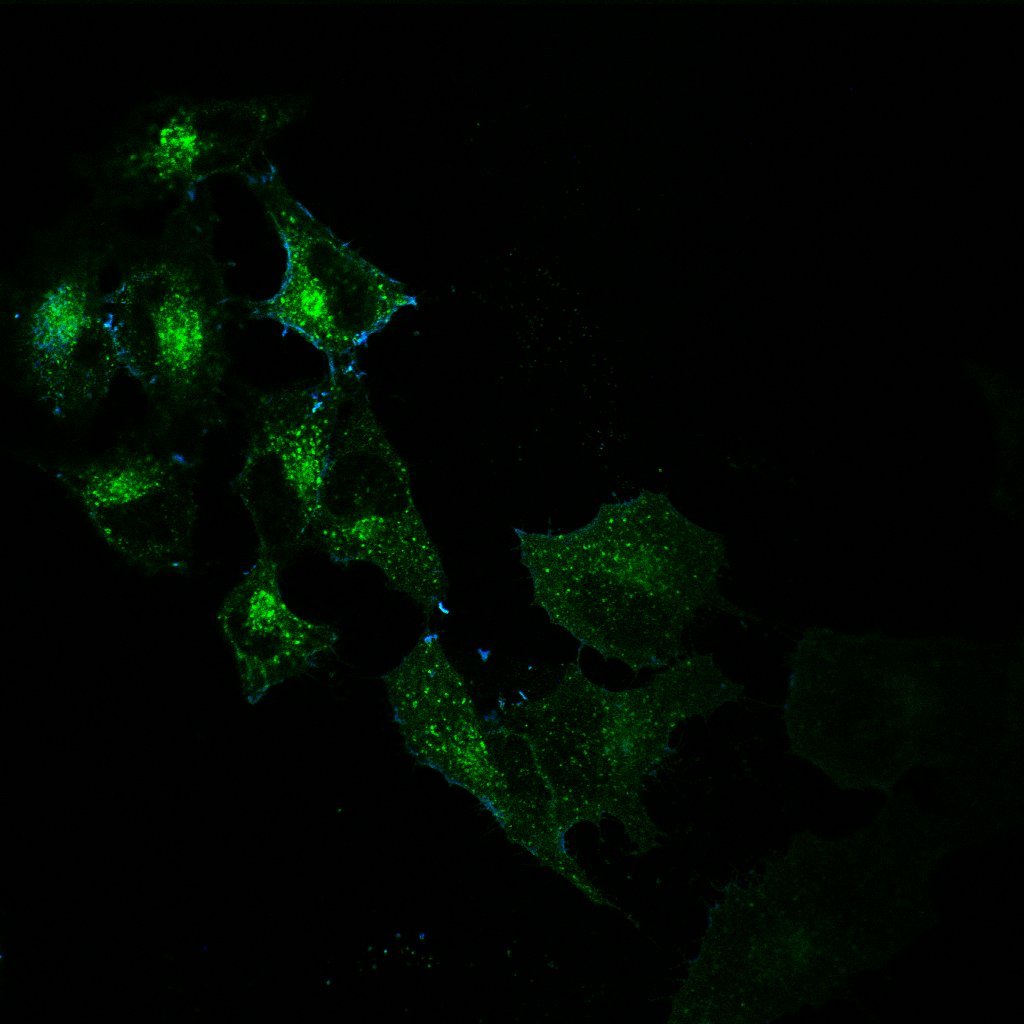

Supplement: Supplemental Information 3 [file peerj-08-8751-s003.zip › Morris et al Data/images for Figure 4/V4Basal1f comp.jpg]

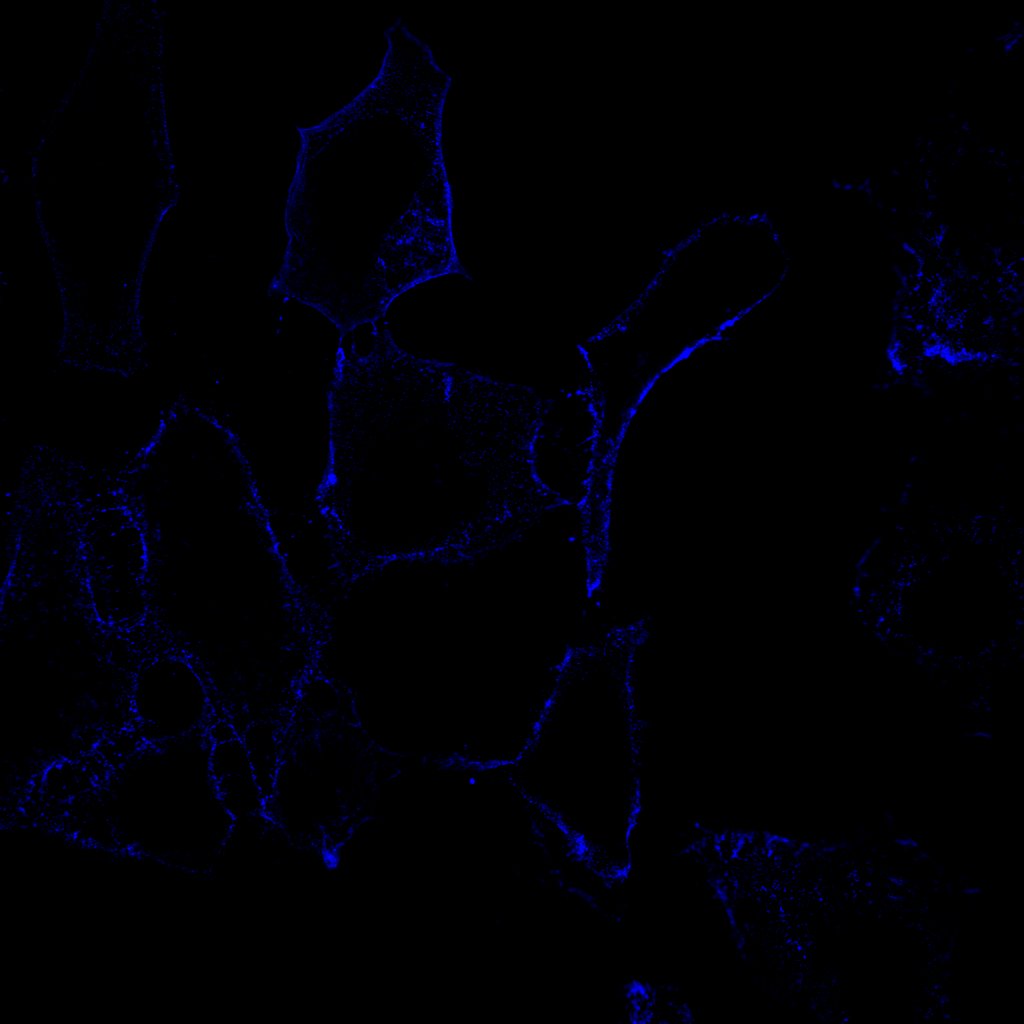

Supplement: Supplemental Information 3 [file peerj-08-8751-s003.zip › Morris et al Data/images for Figure 4/SCRIns1a blue.jpg]

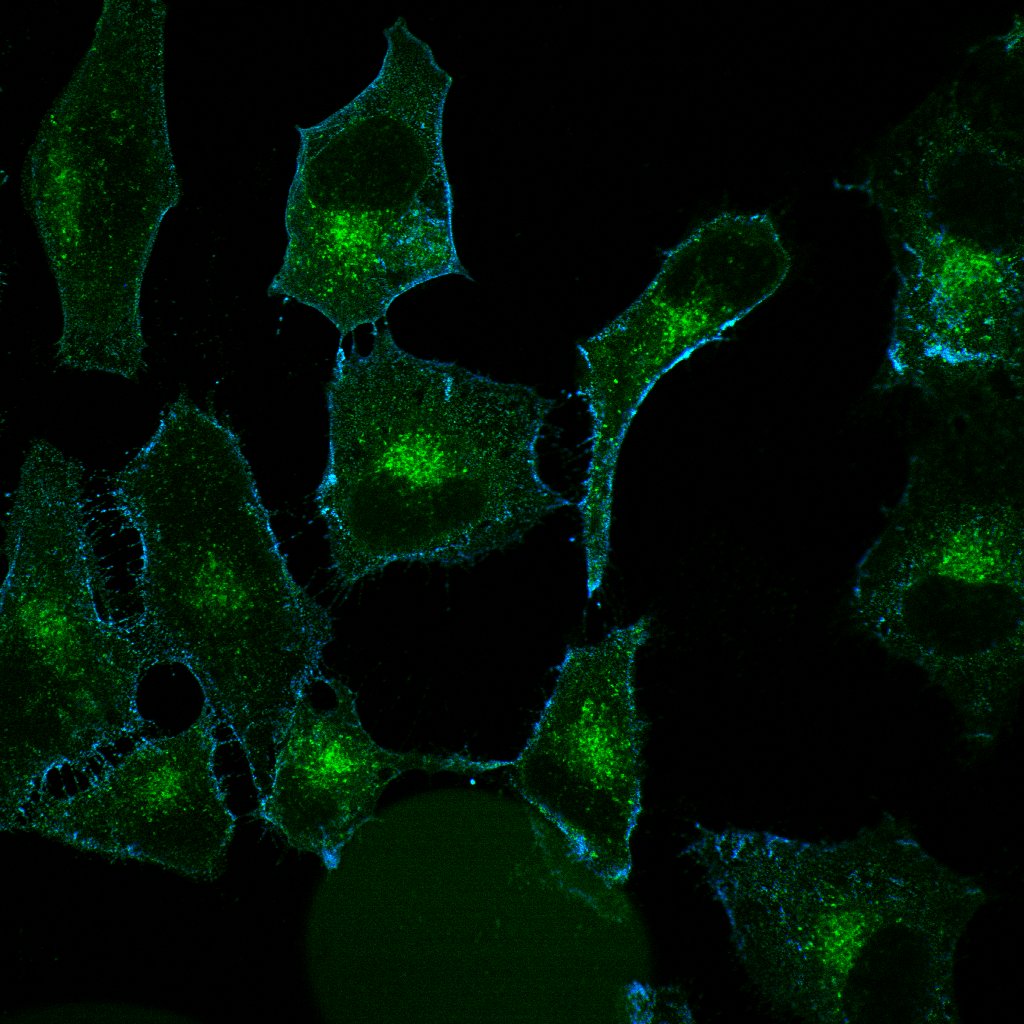

Supplement: Supplemental Information 3 [file peerj-08-8751-s003.zip › Morris et al Data/images for Figure 4/SCRIns1a comp.jpg]

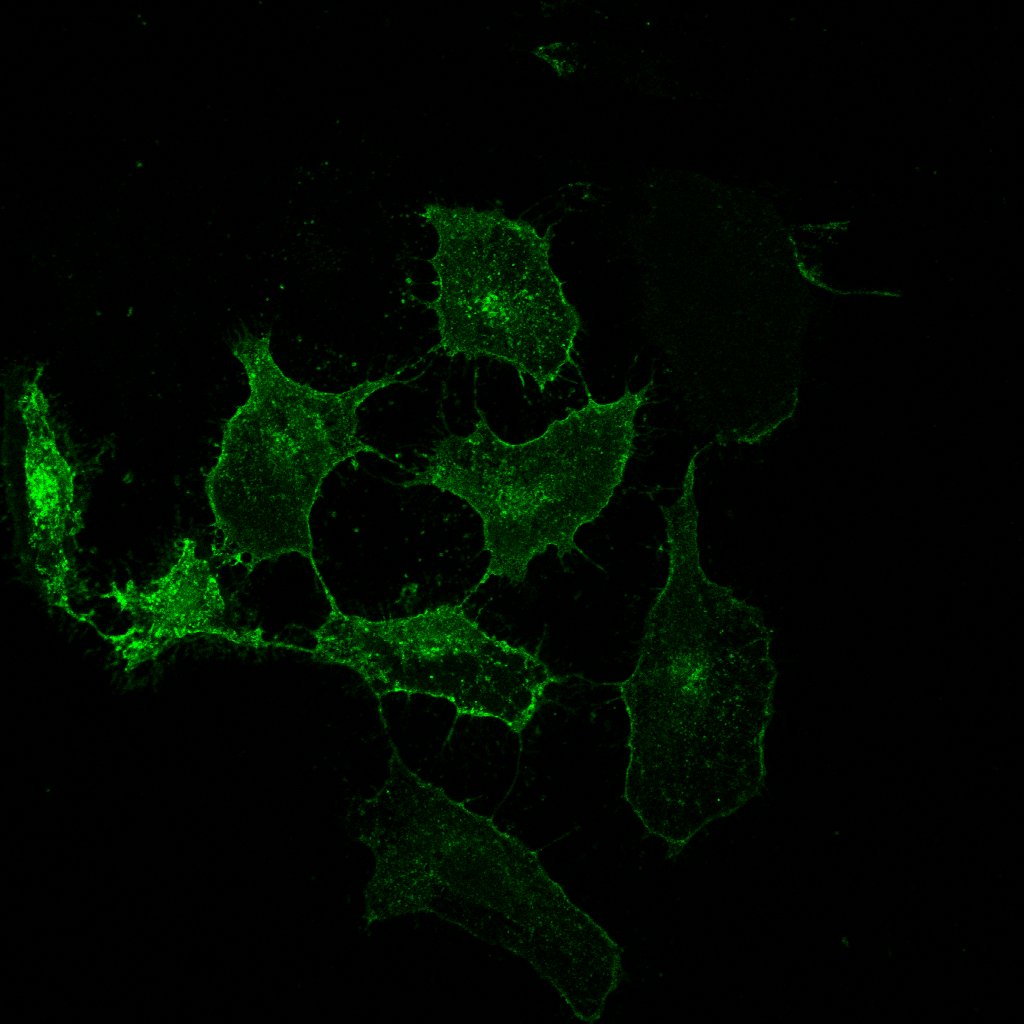

Supplement: Supplemental Information 3 [file peerj-08-8751-s003.zip › Morris et al Data/images for Figure 4/V8Ins1c green.jpg]

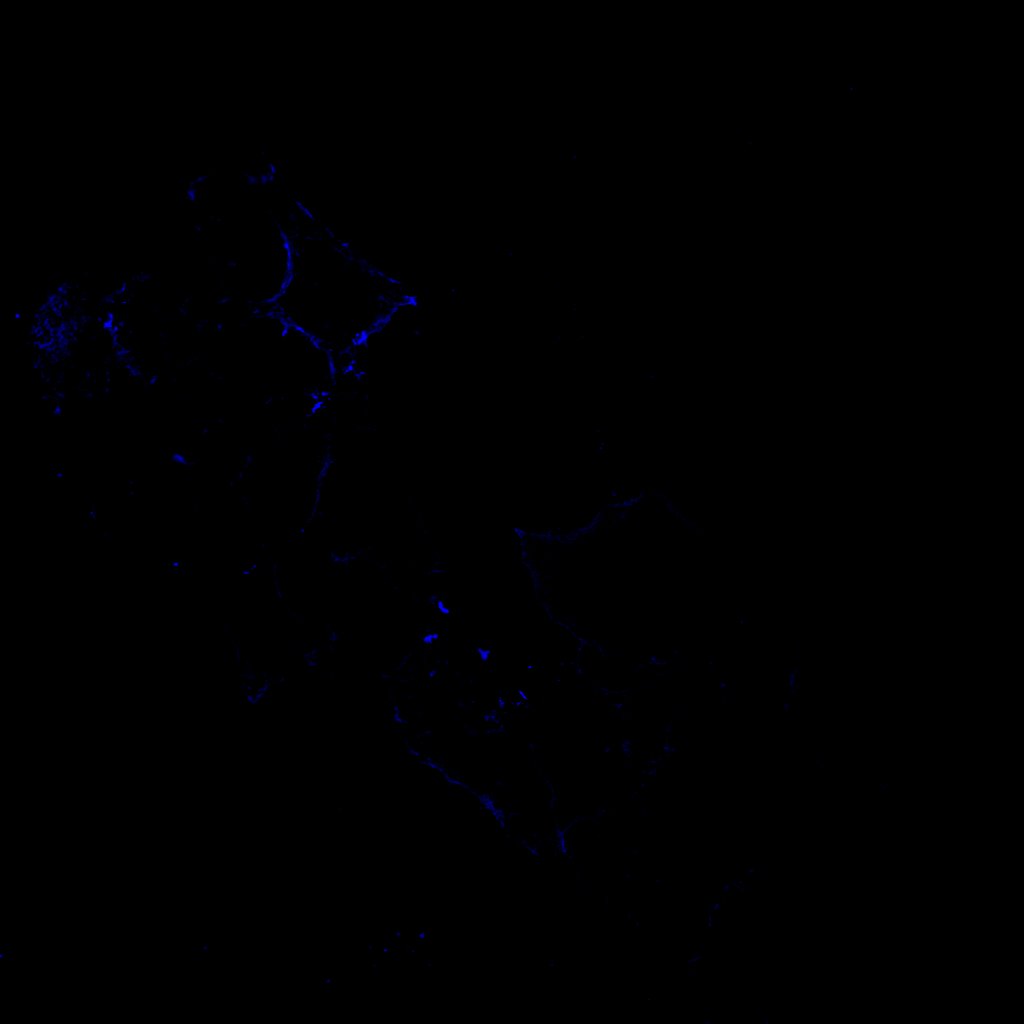

Supplement: Supplemental Information 3 [file peerj-08-8751-s003.zip › Morris et al Data/images for Figure 4/V4Basal1f blue.jpg]

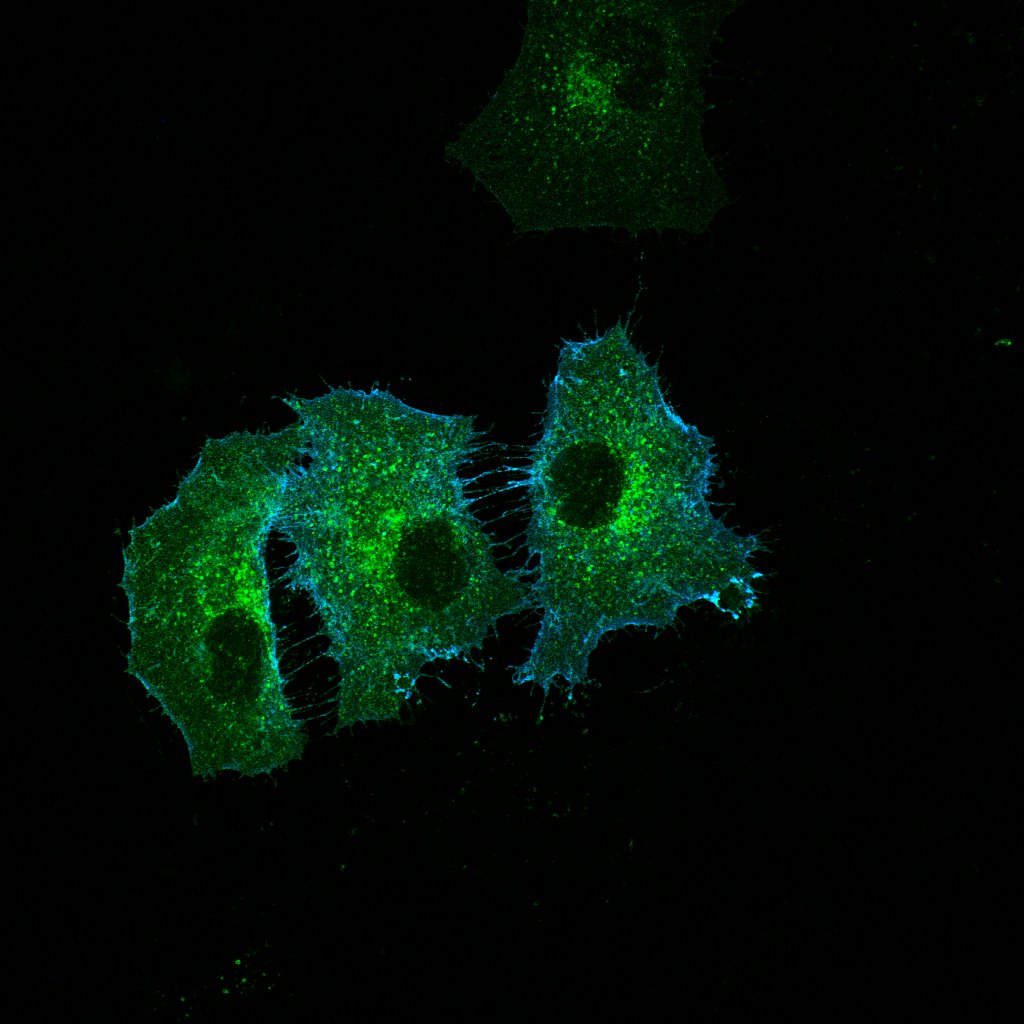

Supplement: Supplemental Information 3 [file peerj-08-8751-s003.zip › Morris et al Data/images for Figure 4/V2Ins1b comp.jpg]

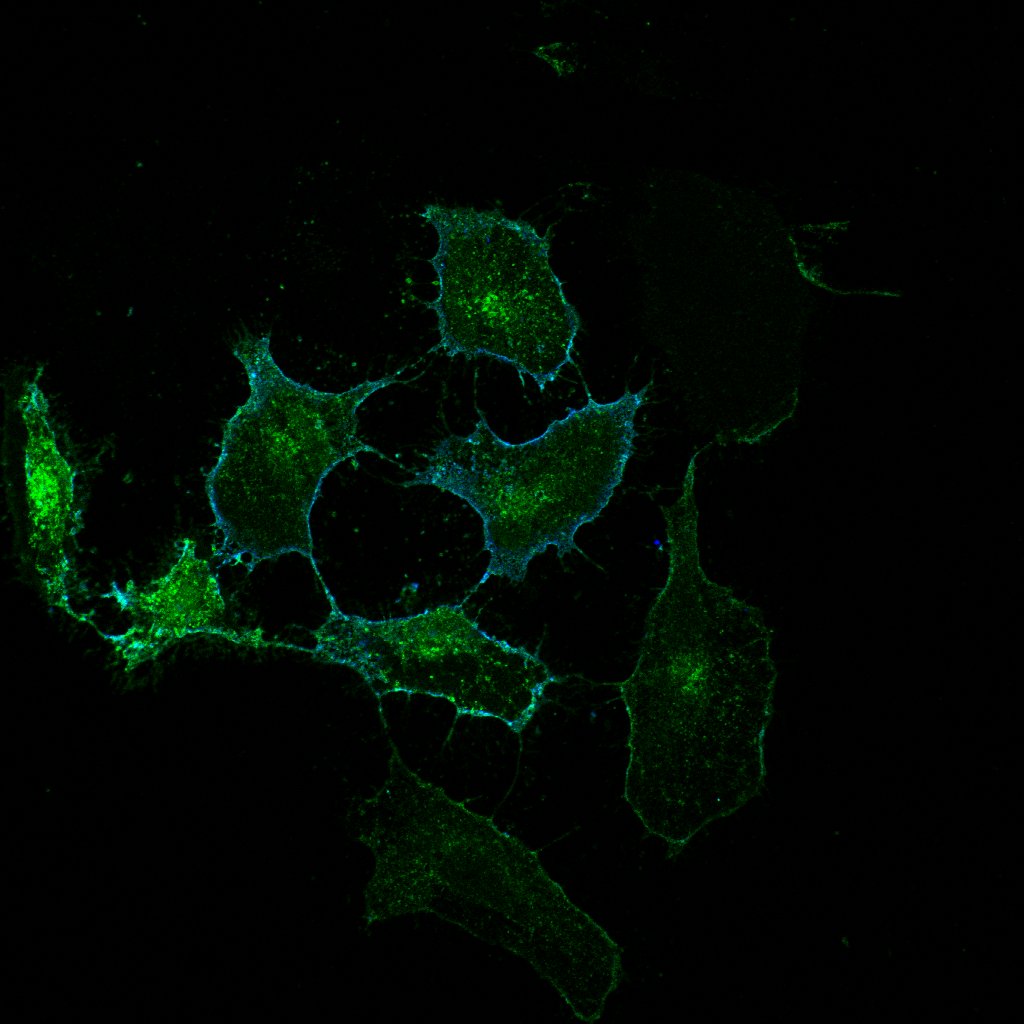

Supplement: Supplemental Information 3 [file peerj-08-8751-s003.zip › Morris et al Data/images for Figure 4/V8Ins1c merge.jpg]

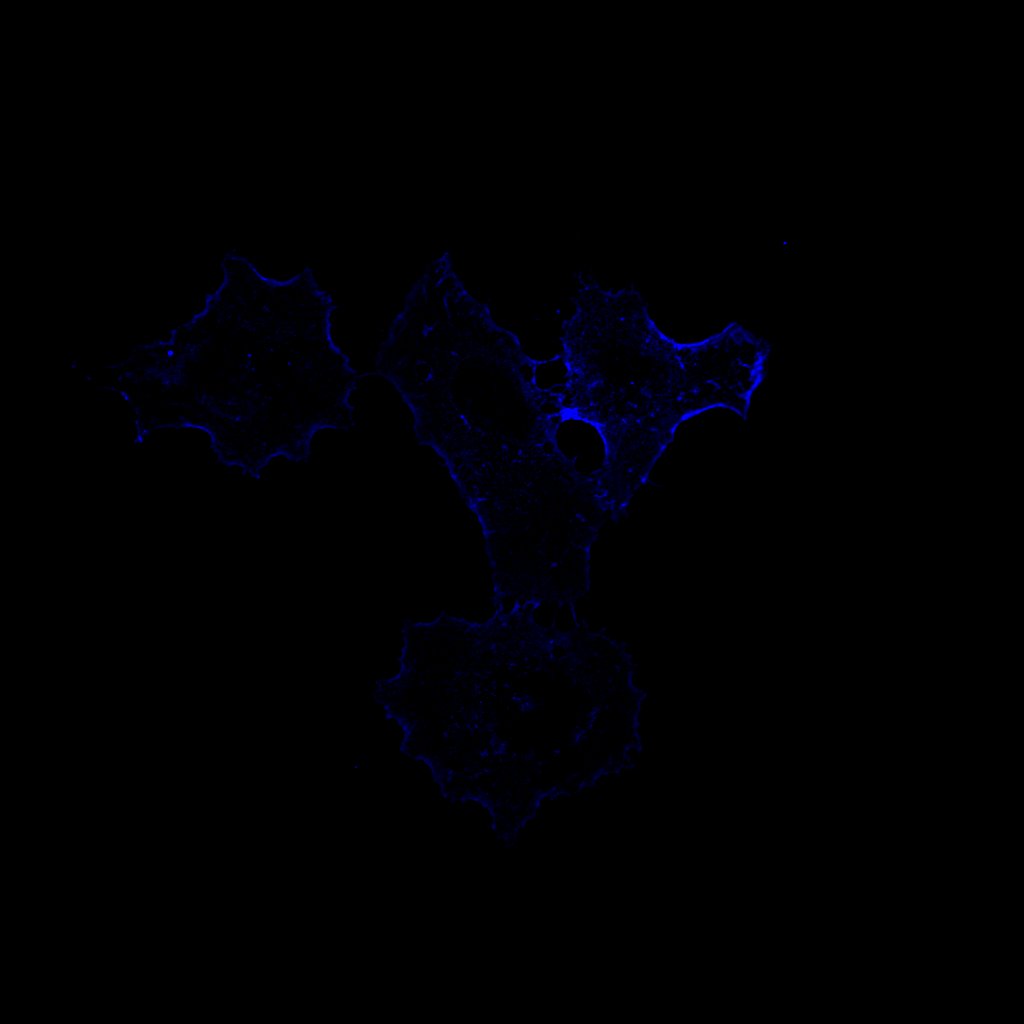

Supplement: Supplemental Information 3 [file peerj-08-8751-s003.zip › Morris et al Data/images for Figure 4/V4Ins1c blue.jpg]

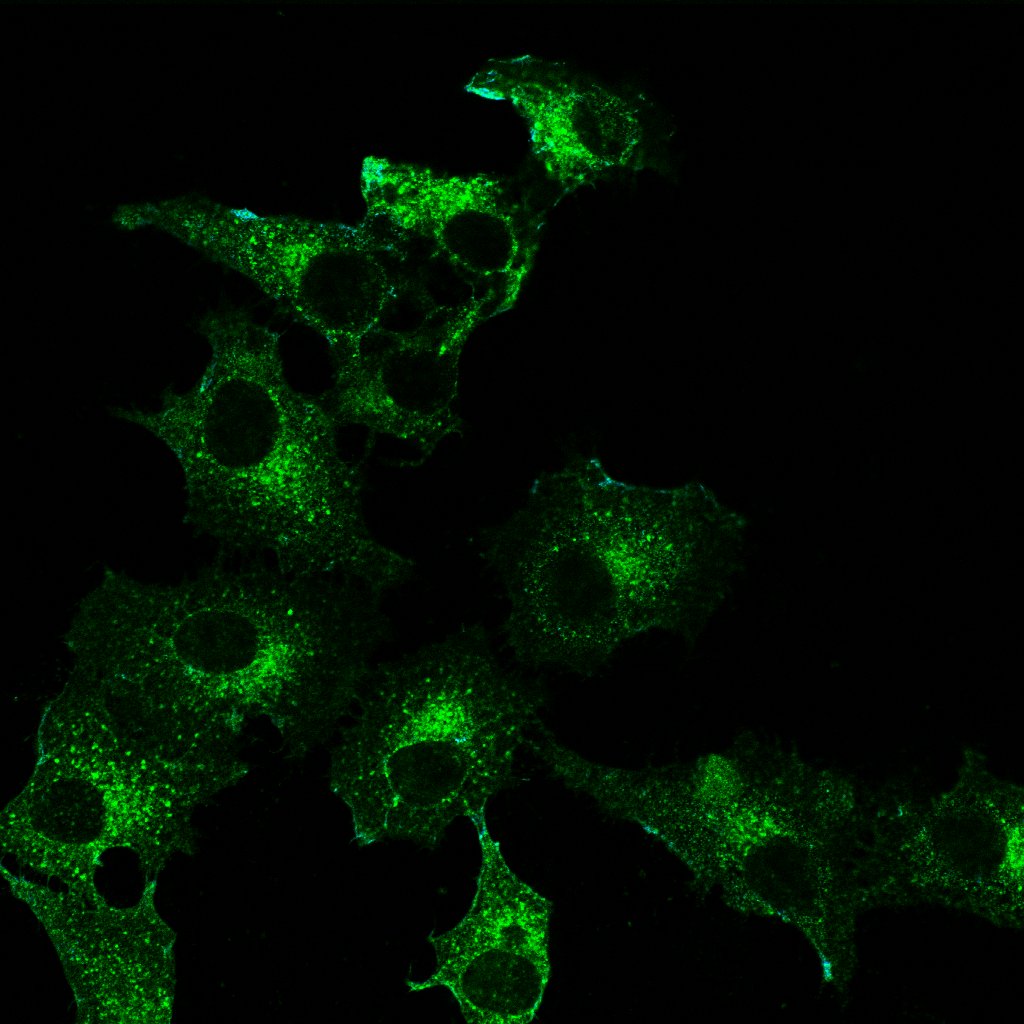

Supplement: Supplemental Information 3 [file peerj-08-8751-s003.zip › Morris et al Data/images for Figure 4/V4basal1c comp.jpg]

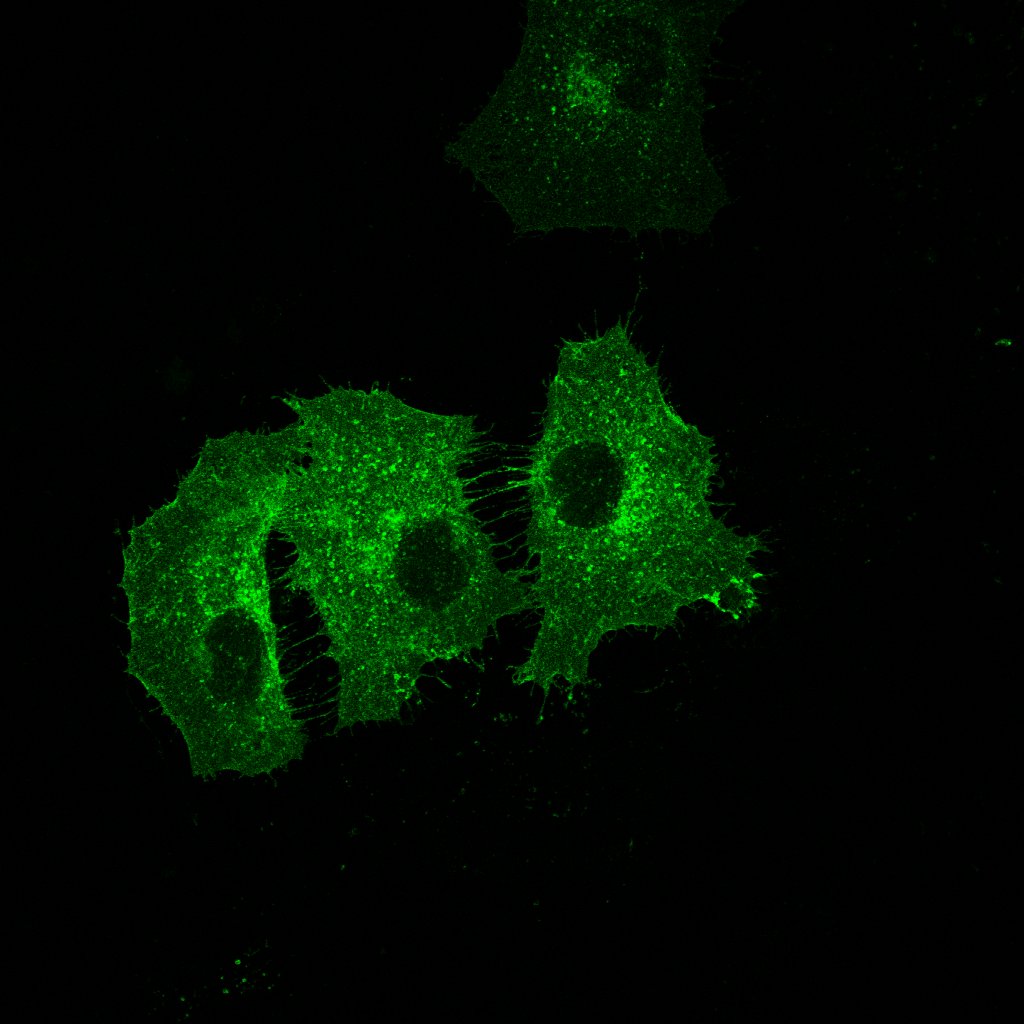

Supplement: Supplemental Information 3 [file peerj-08-8751-s003.zip › Morris et al Data/images for Figure 4/V2Ins1b green.jpg]

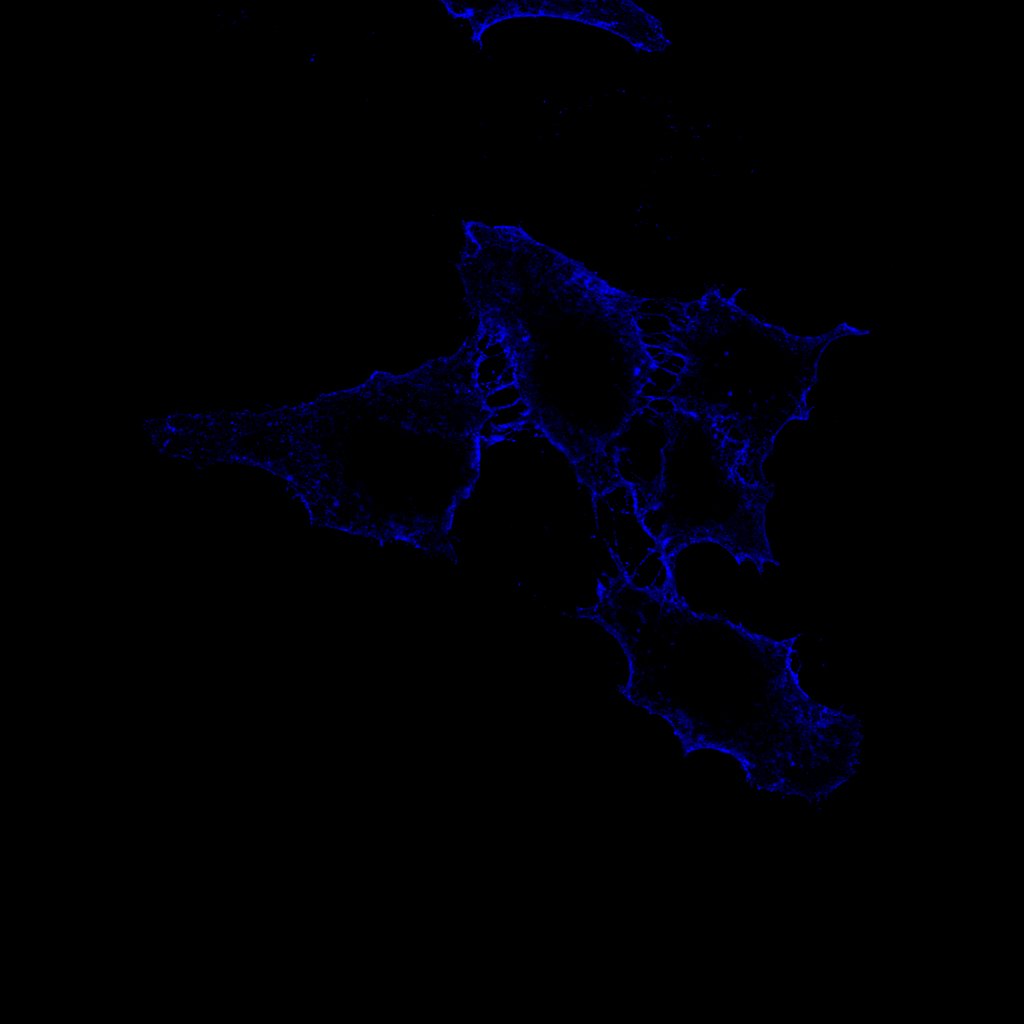

Supplement: Supplemental Information 3 [file peerj-08-8751-s003.zip › Morris et al Data/images for Figure 4/V2basal1a blue.jpg]

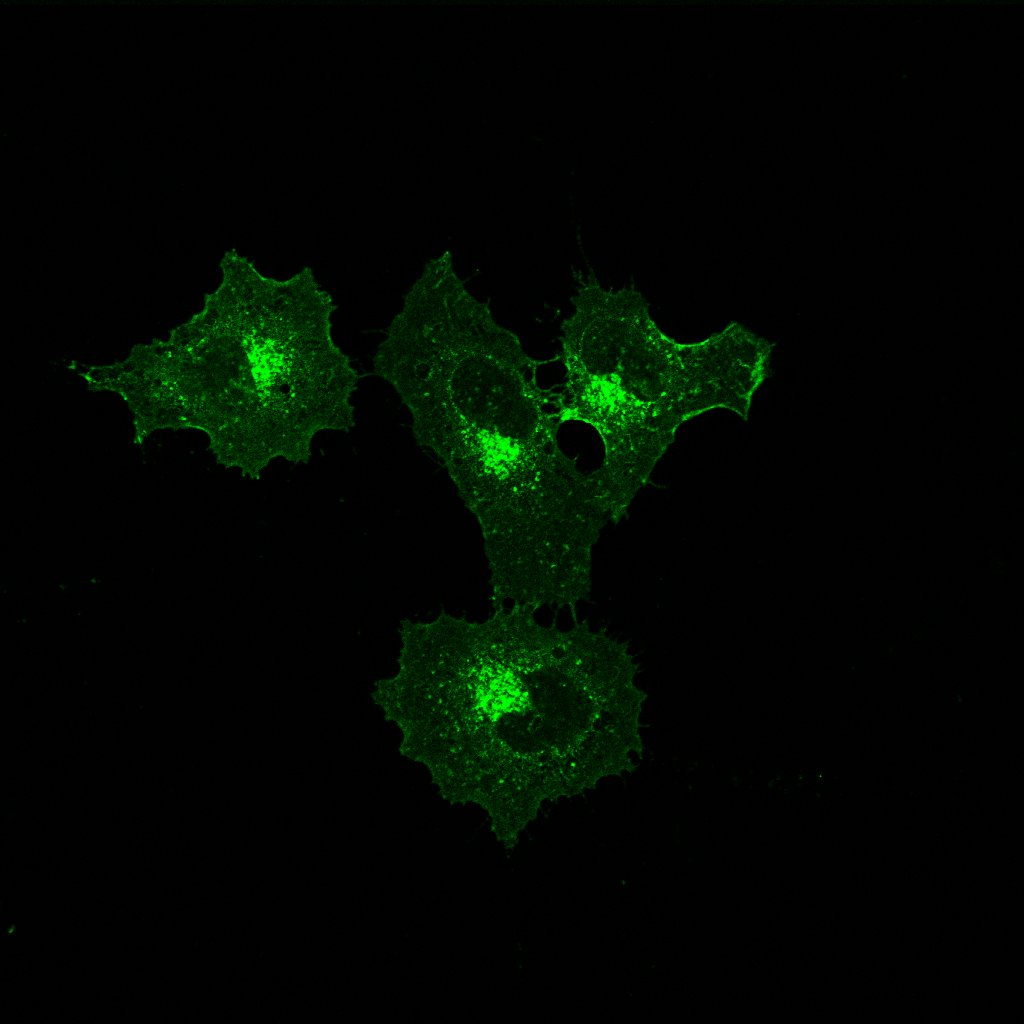

Supplement: Supplemental Information 3 [file peerj-08-8751-s003.zip › Morris et al Data/images for Figure 4/V4Ins1c green.jpg]

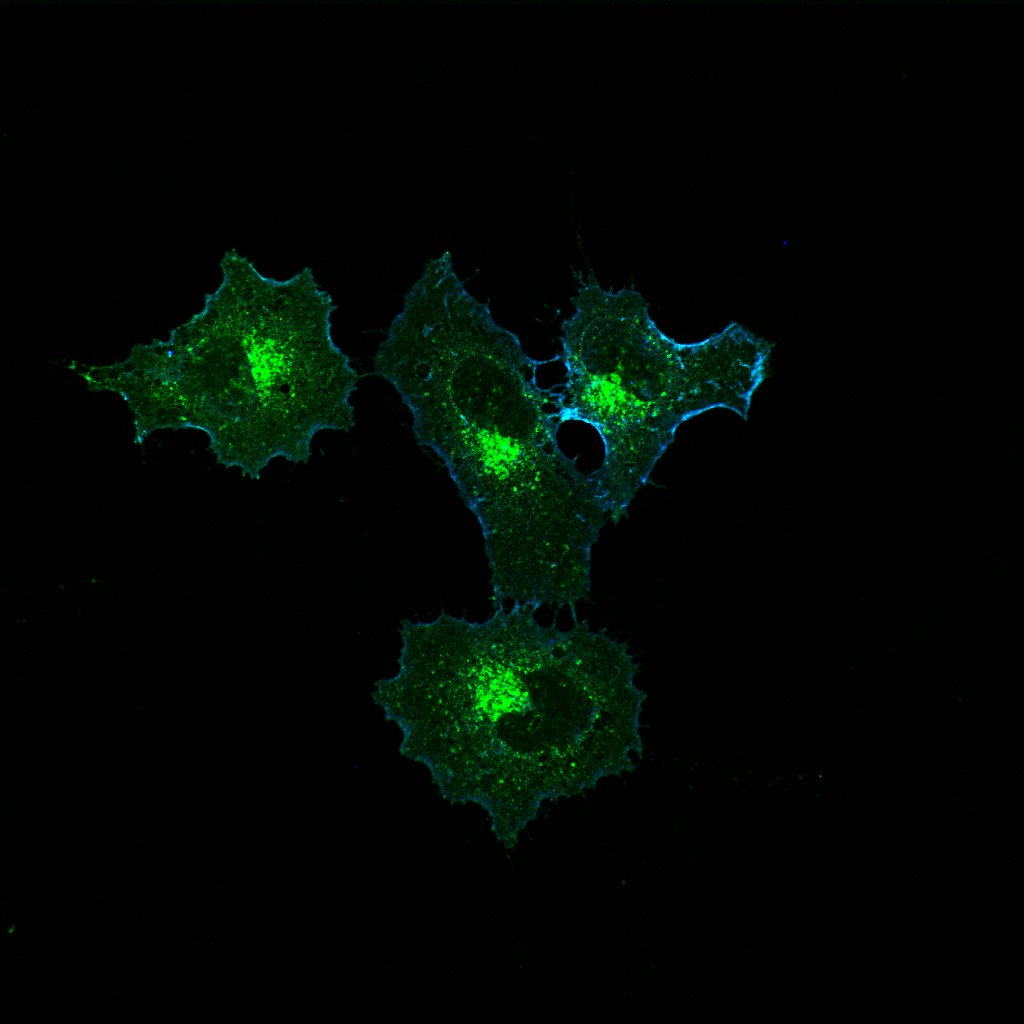

Supplement: Supplemental Information 3 [file peerj-08-8751-s003.zip › Morris et al Data/images for Figure 4/V4Ins1c merge.jpg]

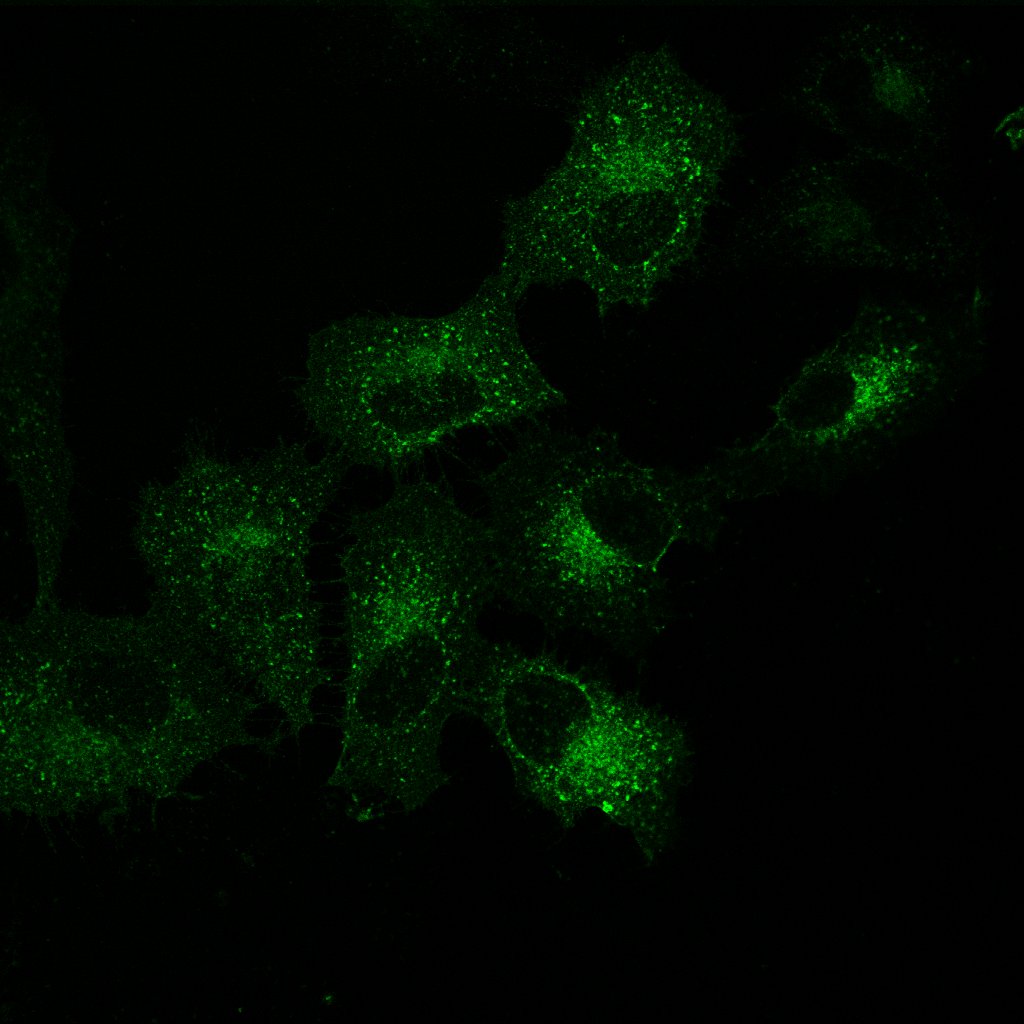

Supplement: Supplemental Information 3 [file peerj-08-8751-s003.zip › Morris et al Data/images for Figure 4/SCRbasal1b0 green.jpg]

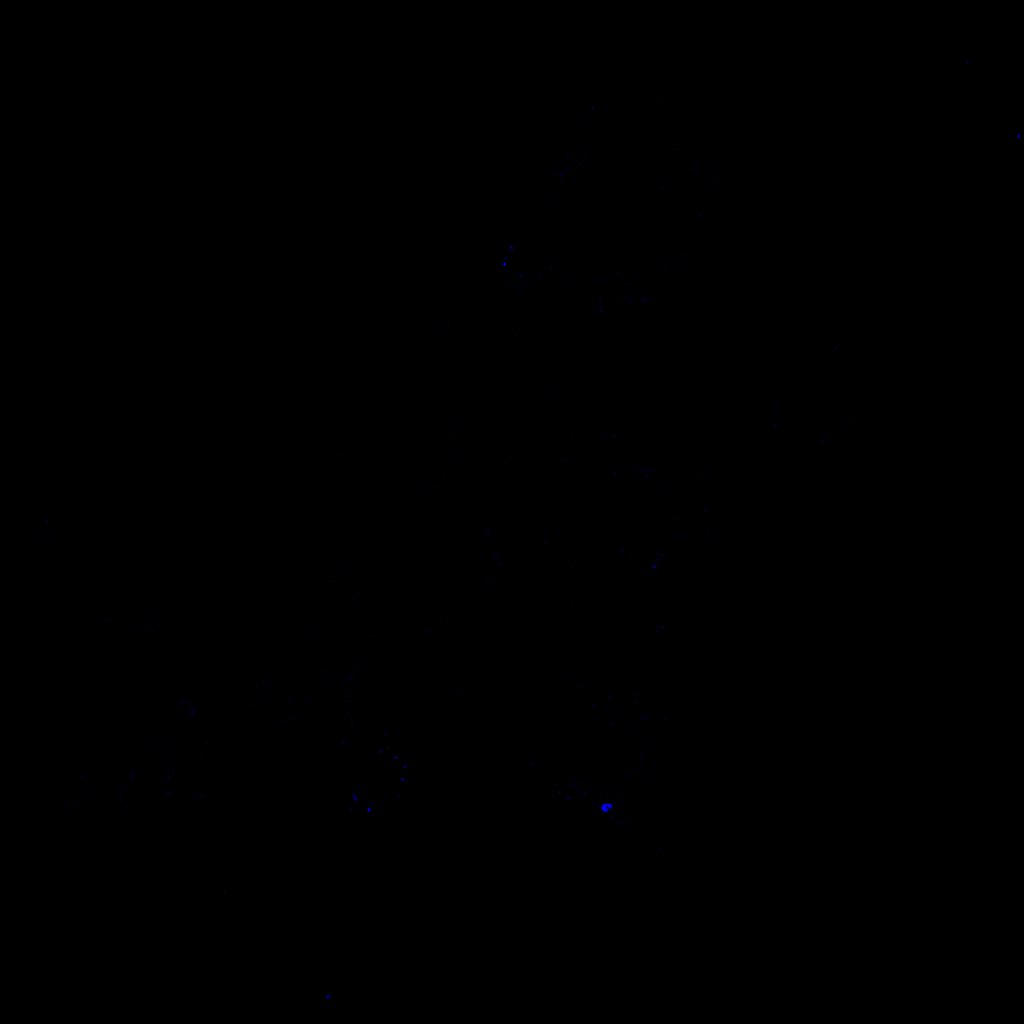

Supplement: Supplemental Information 3 [file peerj-08-8751-s003.zip › Morris et al Data/images for Figure 4/SCRbasal1b0 blue.jpg]

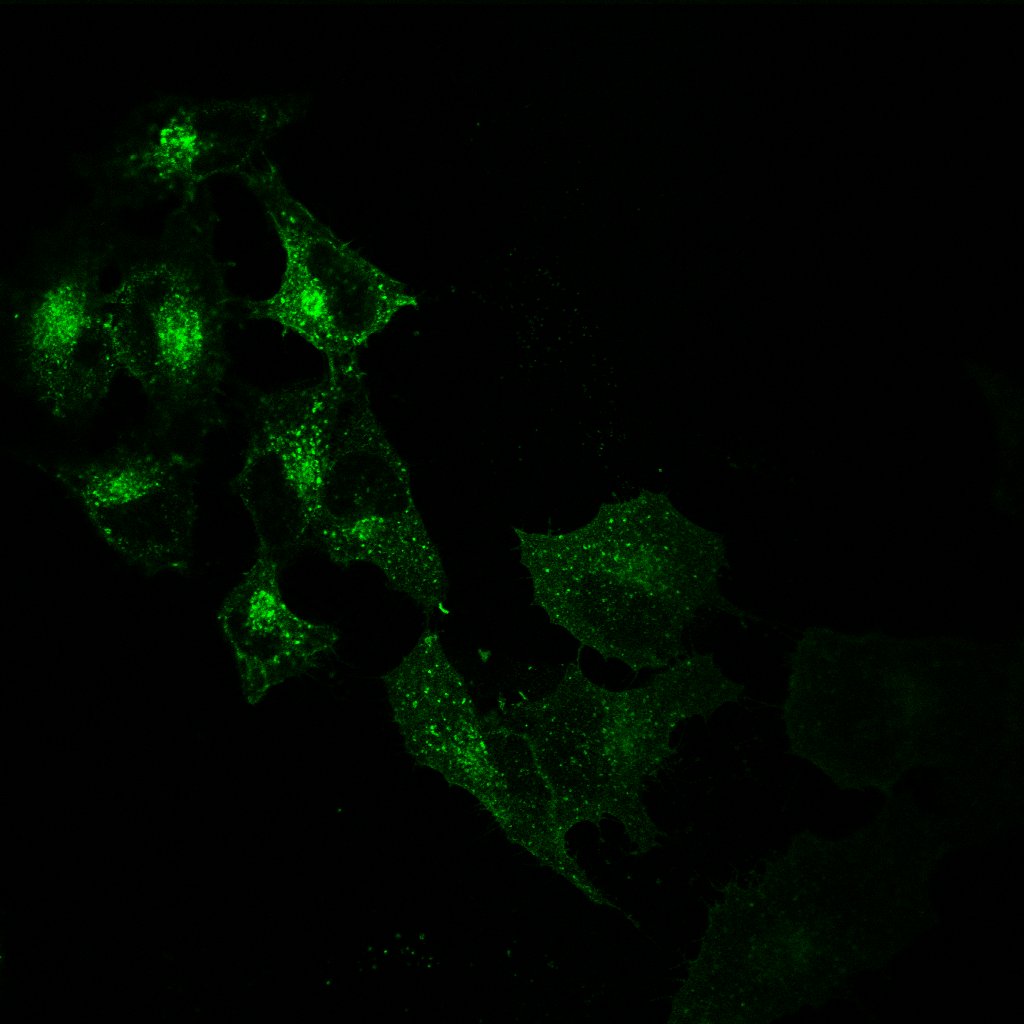

Supplement: Supplemental Information 3 [file peerj-08-8751-s003.zip › Morris et al Data/images for Figure 4/V4Basal1f green.jpg]

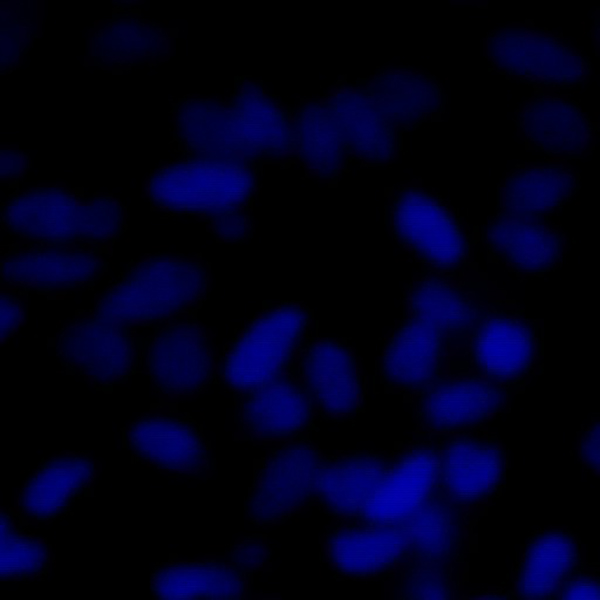

Supplement: Supplemental Information 3 [file peerj-08-8751-s003.zip › Morris et al Data/images for Figure 5/blue.jpg]

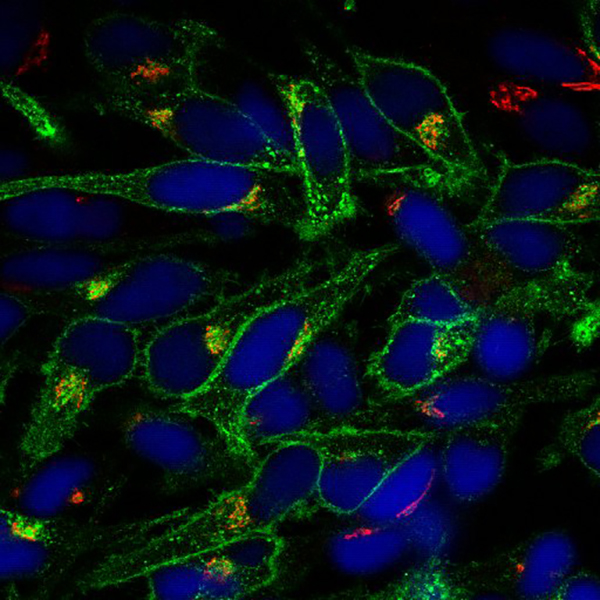

Supplement: Supplemental Information 3 [file peerj-08-8751-s003.zip › Morris et al Data/images for Figure 5/merge.jpg]

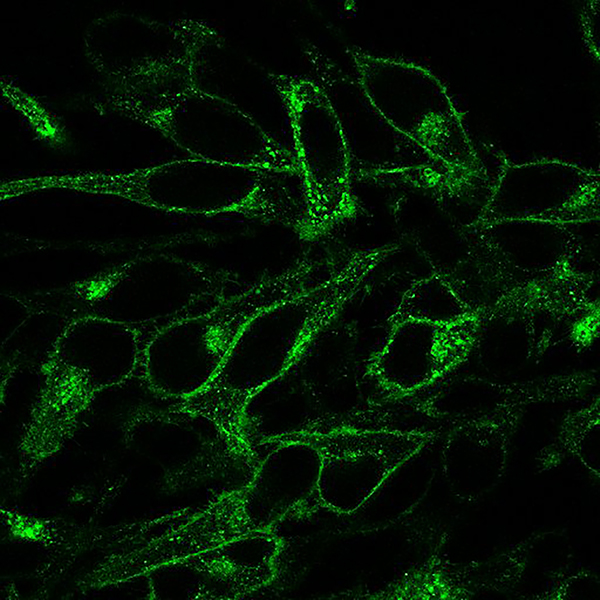

Supplement: Supplemental Information 3 [file peerj-08-8751-s003.zip › Morris et al Data/images for Figure 5/green.jpg]

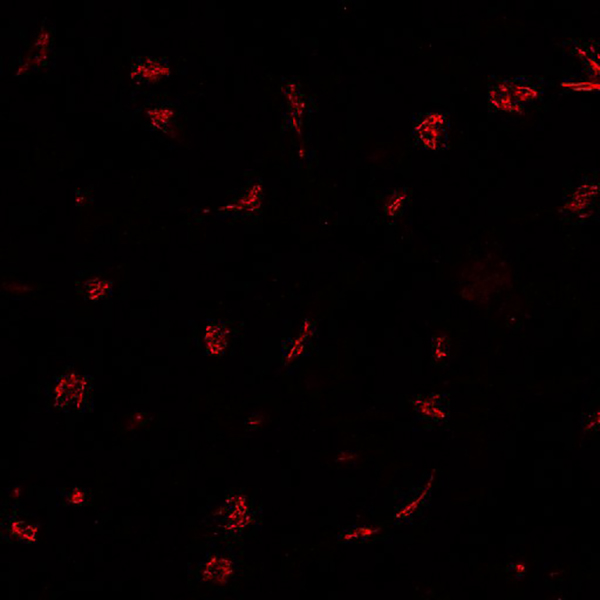

Supplement: Supplemental Information 3 [file peerj-08-8751-s003.zip › Morris et al Data/images for Figure 5/red.jpg]
