# Supplementary material for: Characterisation of GLUT4 trafficking in HeLa cells: comparable kinetics and orthologous trafficking mechanisms to 3T3-L1 adipocytes
Source: PeerJ. 2020 Mar 5;8:e8751. doi: 10.7717/peerj.8751 (PMC7060922; doi:10.7717/peerj.8751)
Supplement: Supplemental Information 3 [file peerj-08-8751-s003.zip › Morris et al Data/images of supp figure 2/HeLa KD 14.11.17.pptx]

## Slide 1
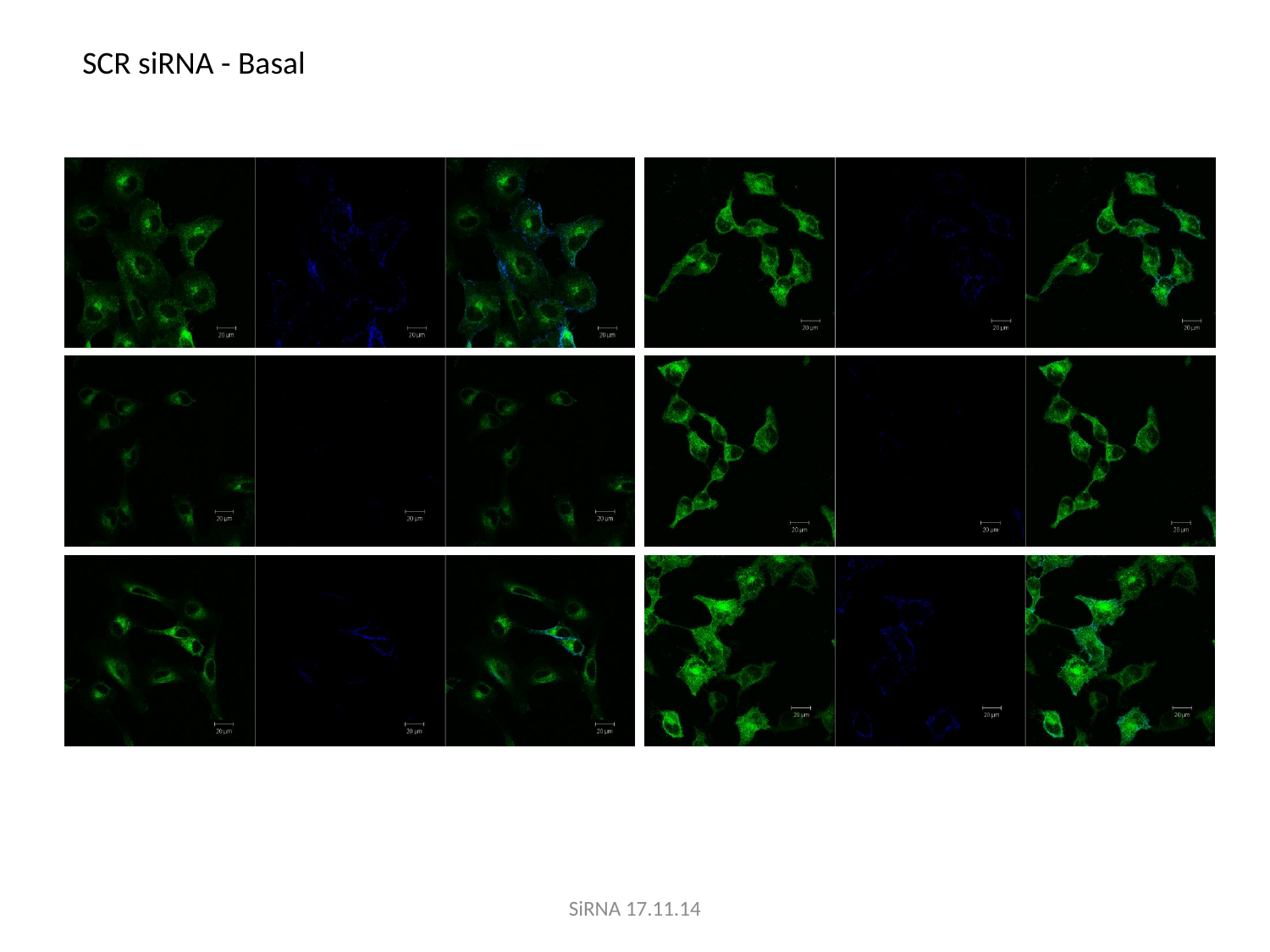

SCR siRNA - Basal
SiRNA 17.11.14

## Slide 2
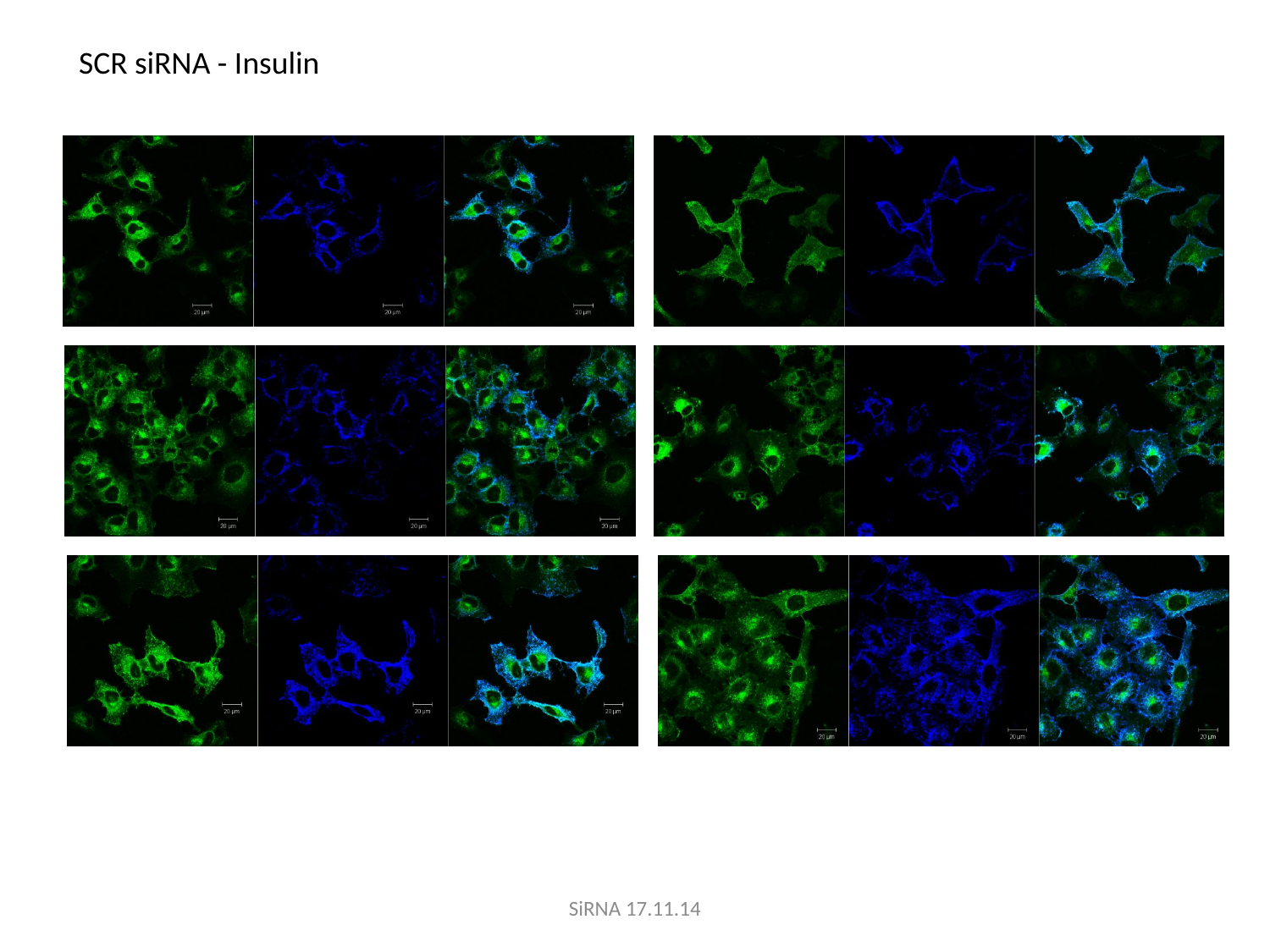

SCR siRNA - Insulin
SiRNA 17.11.14

## Slide 3
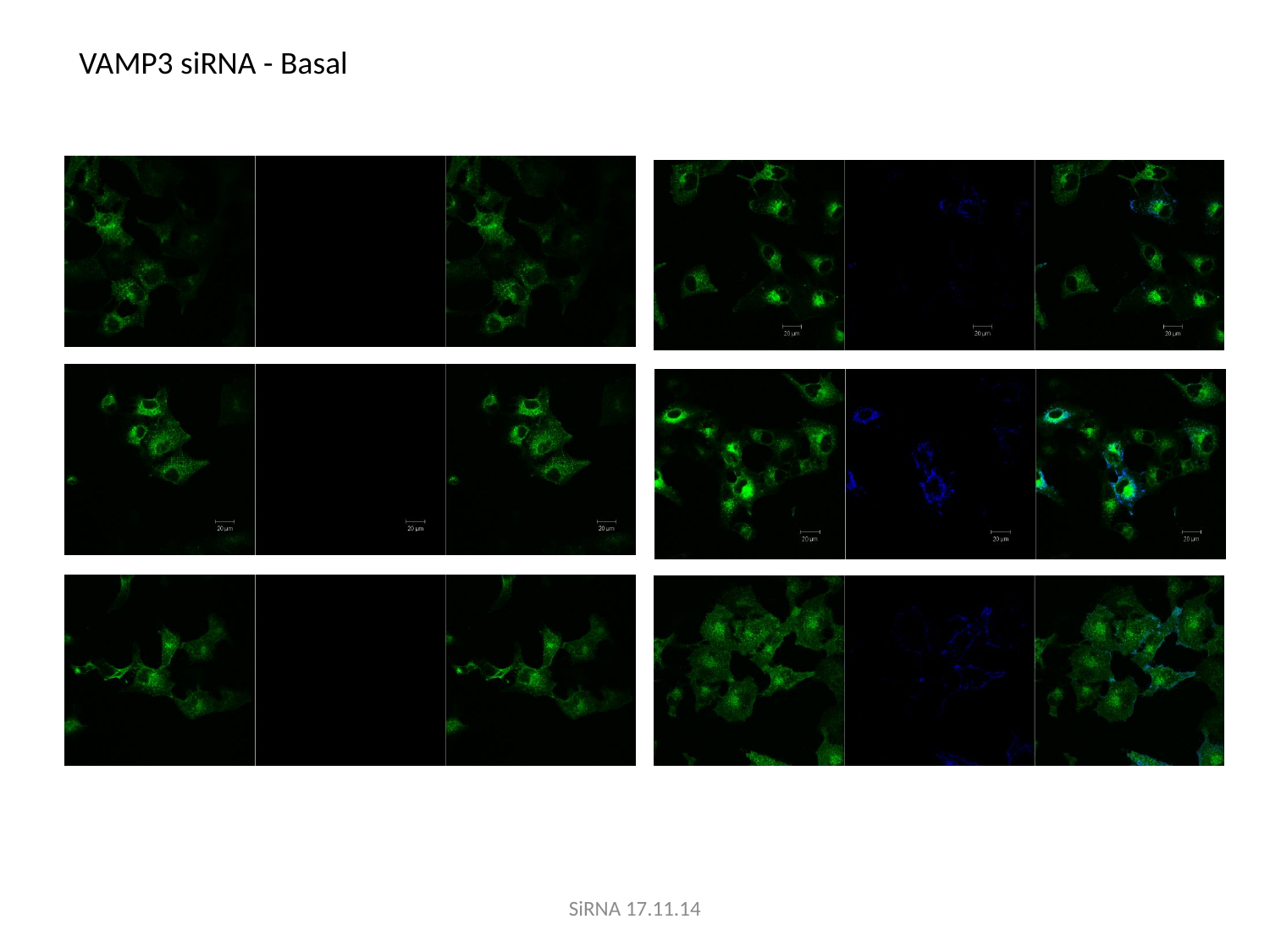

VAMP3 siRNA - Basal
SiRNA 17.11.14

## Slide 4
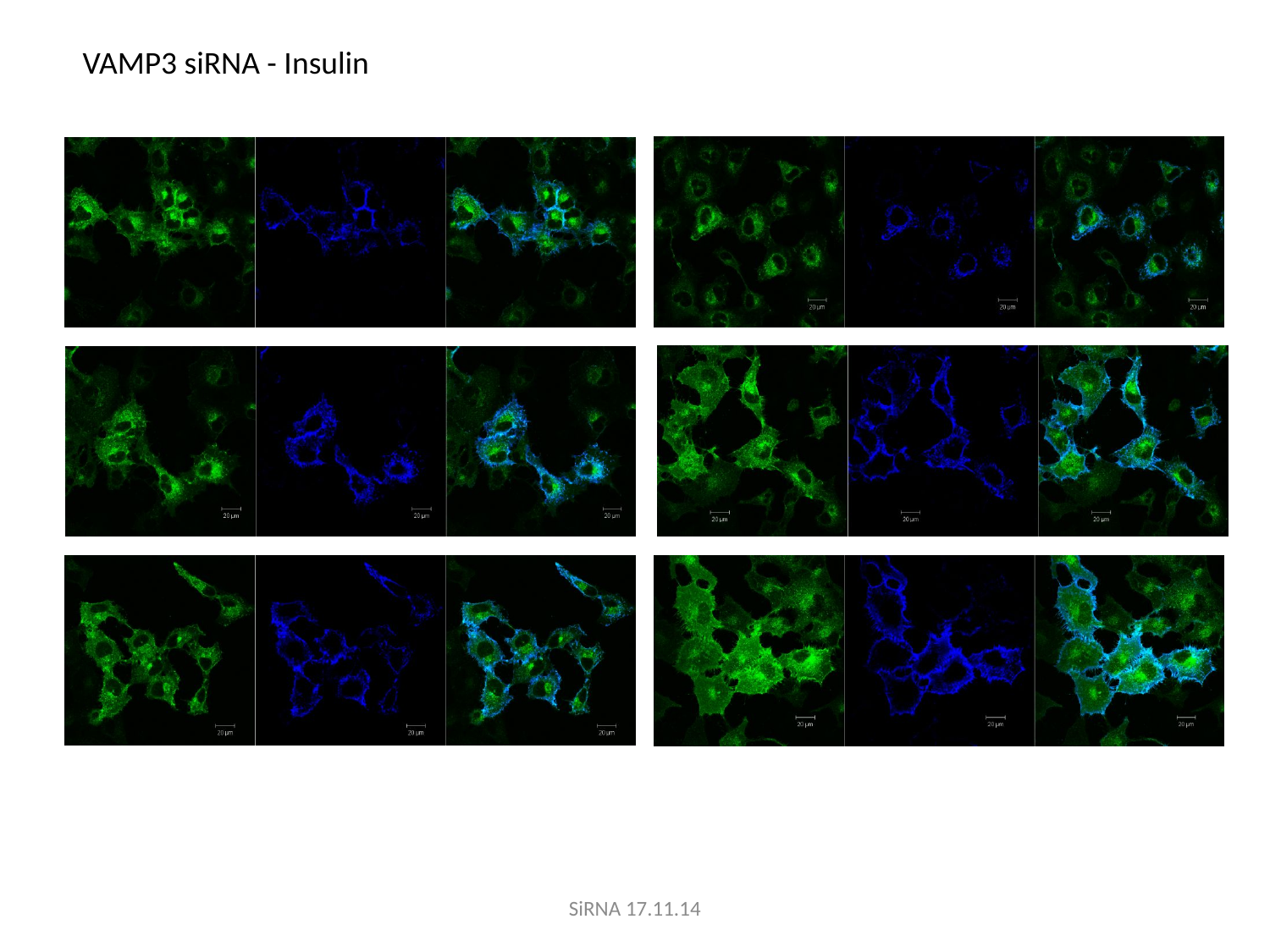

VAMP3 siRNA - Insulin
SiRNA 17.11.14

## Slide 5
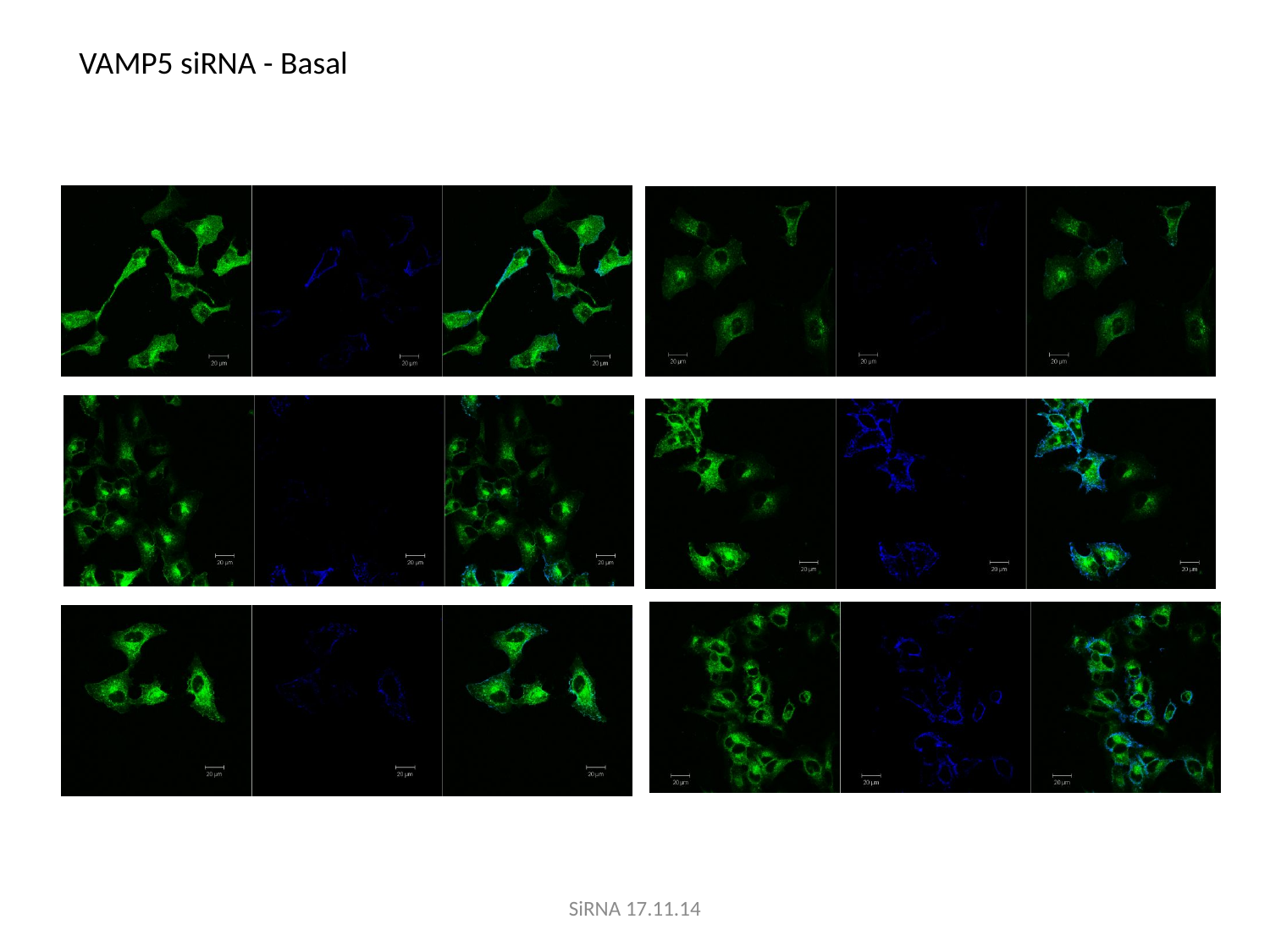

VAMP5 siRNA - Basal
SiRNA 17.11.14

## Slide 6
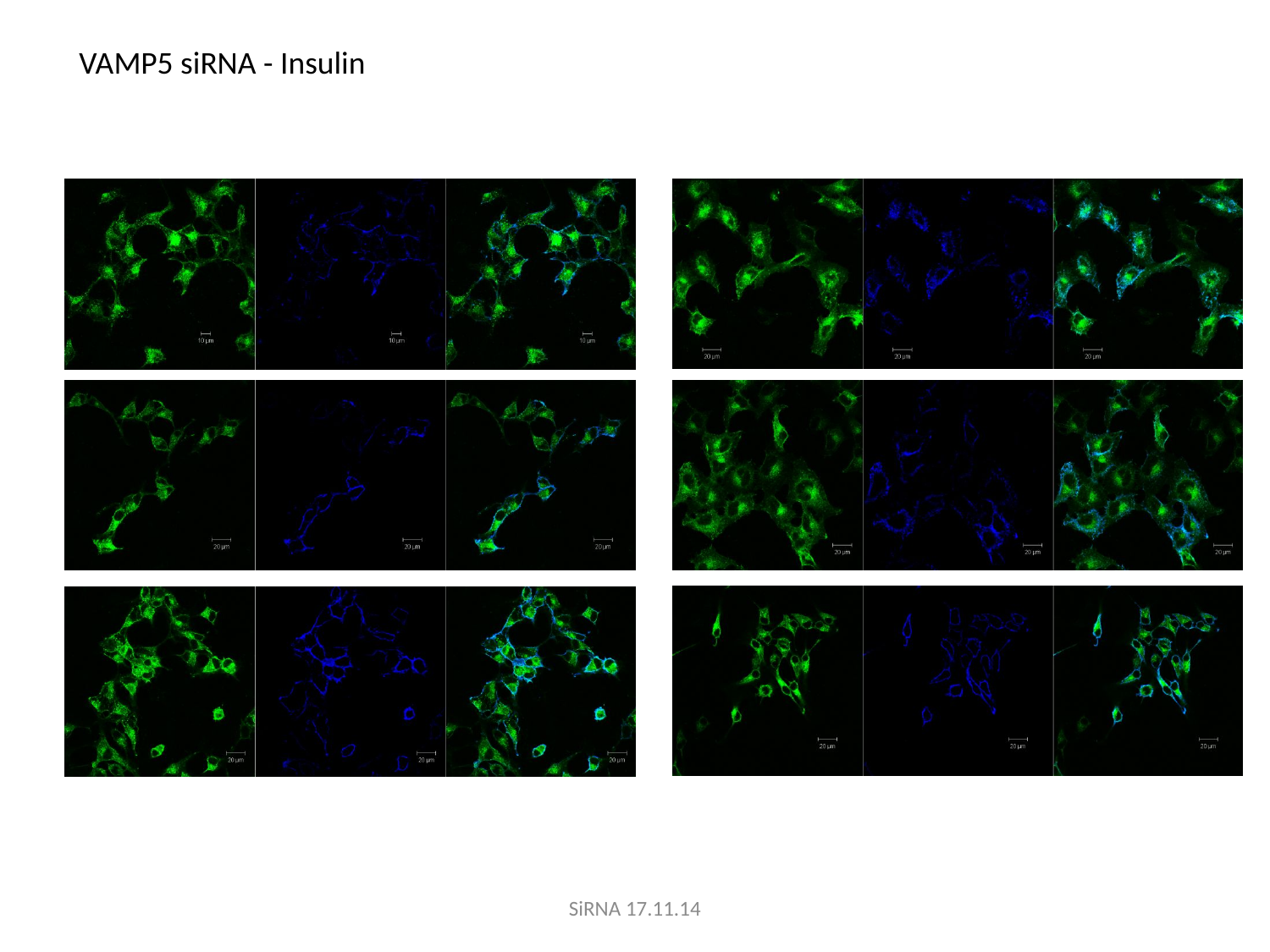

VAMP5 siRNA - Insulin
SiRNA 17.11.14

## Slide 7
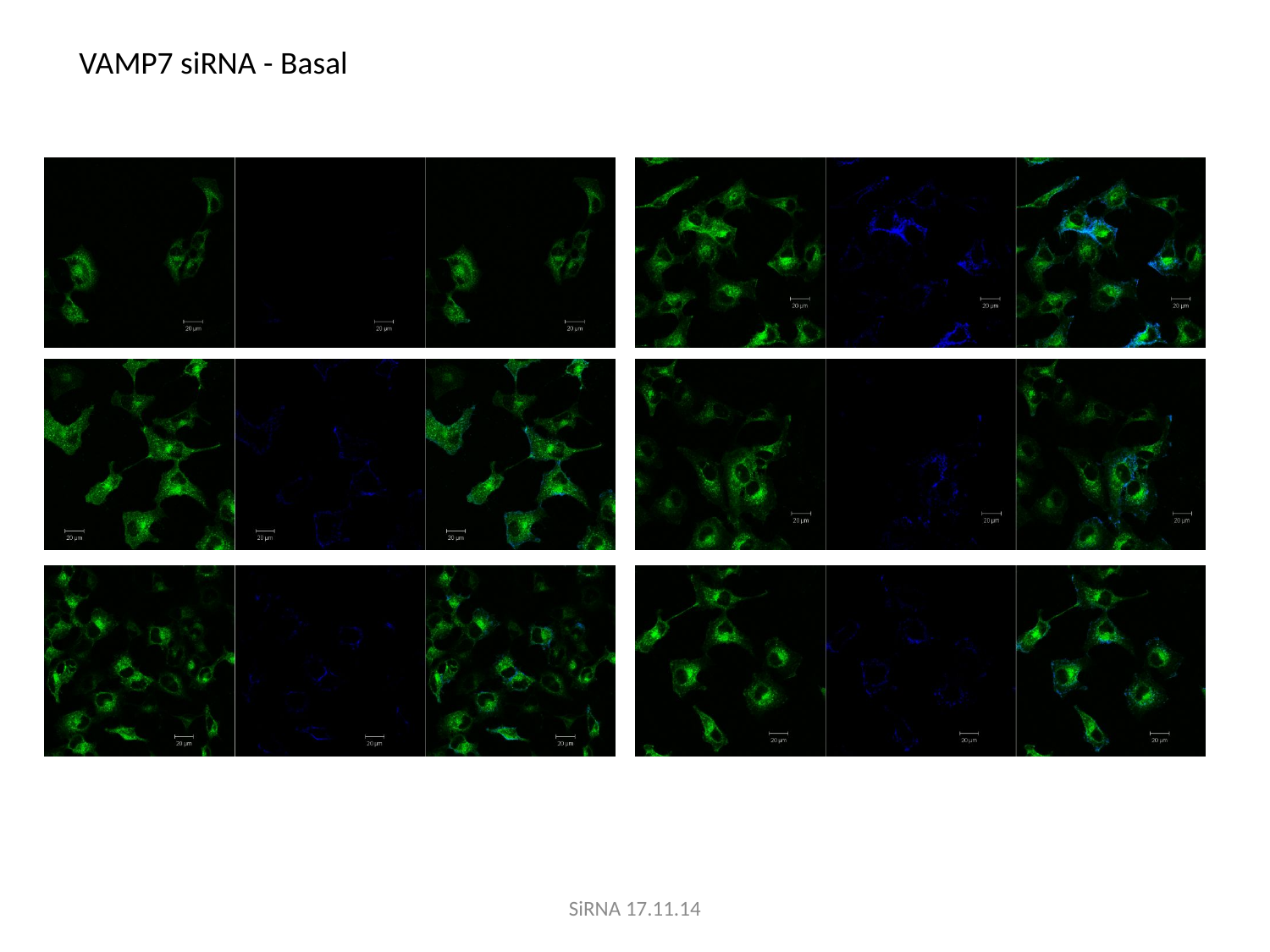

VAMP7 siRNA - Basal
SiRNA 17.11.14

## Slide 8
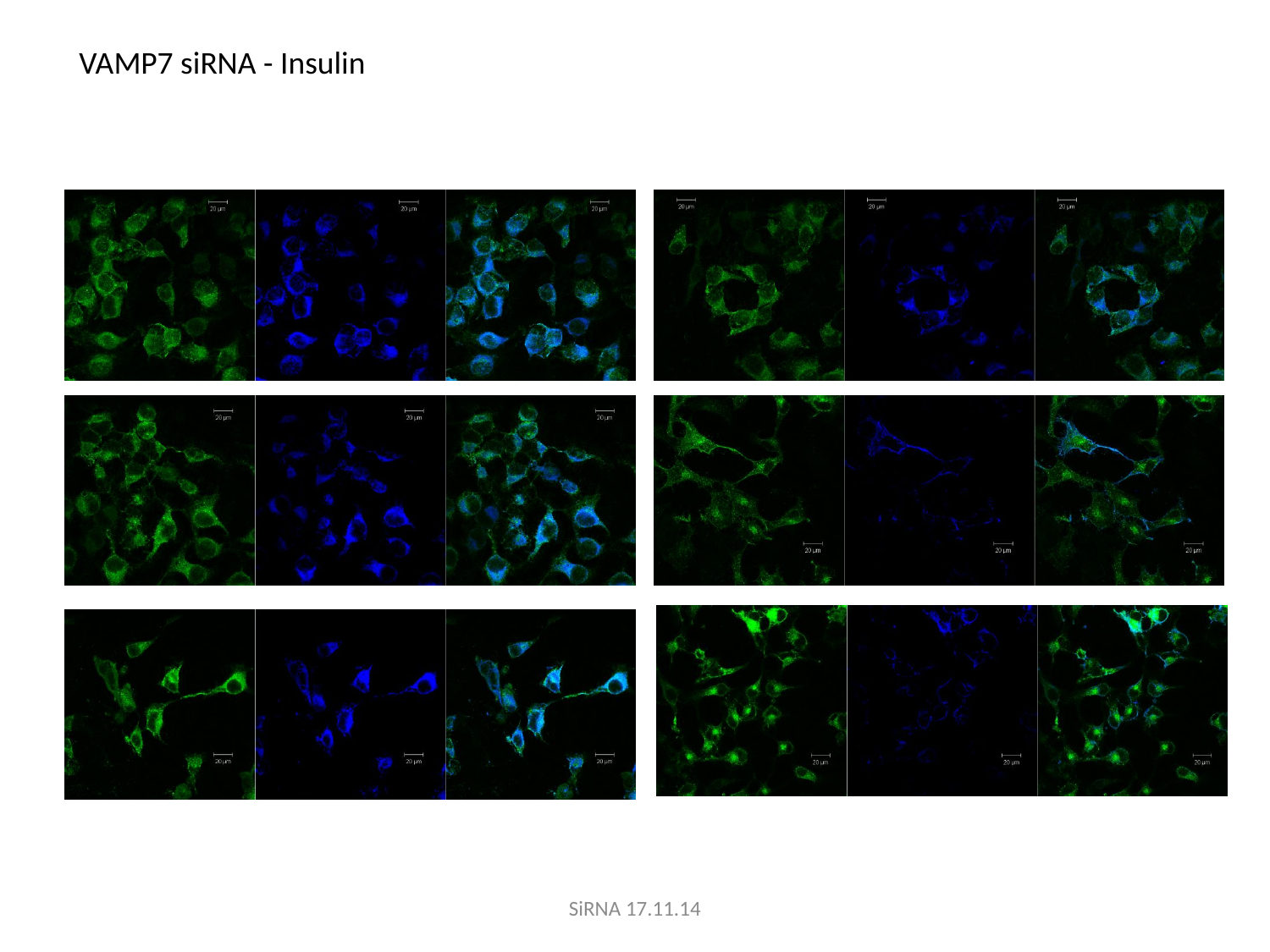

VAMP7 siRNA - Insulin
SiRNA 17.11.14
